# Linking exocellular polysaccharide structures and biosynthetic genes in lactic acid bacteria by combinatorial optimization

**DOI:** 10.1128/aem.00612-26

**Published:** 2026-07-20

**Authors:** Kristian Jensen, Vera Kuzina Poulsen, Paula Gaspar, Pedro Lamosa, Ana Rute Neves, Ahmad A. Zeidan

**Affiliations:** 1 Microbe & Culture Research, Novonesis A/S, Hørsholm, Denmark; 2 Instituto de Tecnologia Química e Biológica António Xavier, Universidade Nova de Lisboa 50106, Oeiras, Portugal; Universita degli Studi di Napoli Federico II, Portici, Italy

**Keywords:** glycosyltransferase, lactic acid bacteria, exocellular polysaccharides, NMR

## Abstract

Lactic acid bacteria produce exocellular polysaccharides (EPSs) that are important in food and health applications, and their functional properties often depend on their chemical structure. These polysaccharides consist of chained repeat units that are assembled stepwise by multiple glycosyltransferases. However, experimental elucidation of EPS structure is time-consuming and labor-intensive, and sequence-based prediction remains limited because the donor and acceptor specificities of glycosyltransferases are difficult to infer from sequence alone. Here, we present a constrained combinatorial optimization framework that integrates data across multiple strains to associate glycosyltransferases in EPS biosynthetic gene clusters with individual sugar residues in EPS repeat units. The framework is constrained by predicted catalytic mechanisms, published experimental evidence, and shared features of glycosyltransferase repertoires and EPS structures across strains. We applied the approach to 10 EPS repeat unit structures elucidated here by nuclear magnetic resonance (NMR) spectroscopy, including four novel structures, together with 15 previously published structures. Using the currently available data set, we could putatively and uniquely assign 42 of 144 glycosyltransferases (29%) to a single repeat-unit residue and determine donor specificity for 60 of 144 glycosyltransferases (41%). This work improves the mapping of EPS genotype-phenotype relationships in lactic acid bacteria and contributes to advancing genome-based prediction of EPS structure.

**IMPORTANCE:**

The structure of exocellular polysaccharides produced by lactic acid bacteria is important for their functional roles in food and health applications but remains difficult to predict from genome sequence because glycosyltransferase specificity is hard to infer and structural characterization is slow. By combining new exocellular polysaccharide (EPS) repeat unit structures with a constrained multi-strain computational framework, we obtain a partial mapping between *eps* genes and polysaccharide structure. This work provides a foundation for genome-based selection of lactic acid bacteria with desirable EPS-related properties.

## INTRODUCTION

Lactic acid bacteria (LAB) are widely used in the food and nutrition industries, serving as starter cultures in fermented foods and as probiotics. A key characteristic of many LAB that makes them valuable in food production and confers health benefits is their ability to produce exocellular polysaccharides, which can either be attached to the cell envelope (capsular polysaccharide) or secreted into the surrounding environment (exocellular polysaccharide) (1). Here, the term exocellular polysaccharide (EPS) is used to refer to these polysaccharides, whether they are capsular or extracellular. In the production of yogurt, cheese, and their plant-based analogs, the use of EPS-producing LAB strains can lead to improved texture of the final product (2–6). Although the benefits from using LAB often depend on the amount of EPS that is produced, evidence suggests that some applications are also sensitive to the chemical structure of the produced EPS, including monomer composition, molecular weight, degree of branching, and glycoside linkage patterns (1, 7). Consequently, studies on the effects of EPS should ideally account for its structure. Since experimental elucidation of EPS structure can be both time-consuming and costly, the ability to infer EPS structure based on genomic data would be highly advantageous.

LAB have been shown to produce two classes of EPS: homopolymeric EPS and heteropolymeric EPS (1). Most homopolymeric EPS is produced by a sucrase-dependent pathway, relying on a single enzyme (sucrase) to produce large amounts of homopolymeric exocellular polysaccharide by breaking down sucrose extracellularly (8). Recently, a sucrose-independent homopolymeric EPS in *Lactobacillus johnsonii* was found to be produced using an enzyme with high similarity to a bactoprenol glycosyltransferase (9). Although a range of different homopolymeric polysaccharides exists, their simple structure and the monogenic nature of the synthesis pathway allow the link between genome content and EPS structure to be reduced to the functional characterization of a single enzyme. In contrast, this study will focus on the association between genes and the structure of heteropolymeric EPS, which are produced via the Wzy-dependent pathway.

### Wzy-dependent EPS biosynthesis

**Fig 1:**
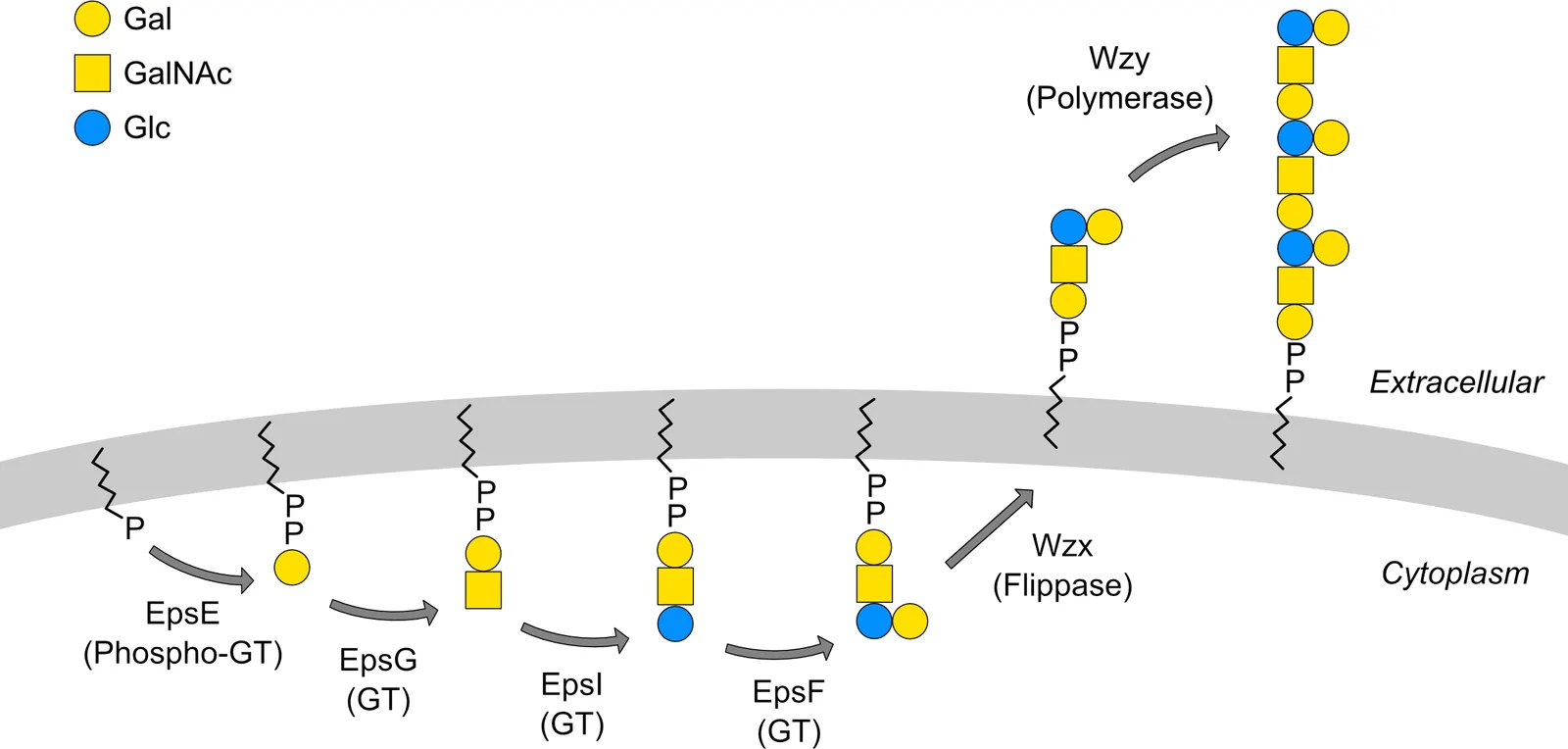
Overview of the EPS repeat unit synthesis and polymerization by the Wzy-dependent pathway. The repeat unit structure and protein names are exemplified by the *Streptococcus thermophilus* Sfi6 strain. Gal, galactose; GalNAc, N-acetylgalactosamine; Glc, glucose; and GT, glycosyltransferase.

The Wzy-dependent pathway is encoded in a gene cluster, here referred to as an *eps* cluster, which can be found either on the chromosome or on a plasmid, depending on the LAB species (1). Generally, *eps* clusters consist of regions of well-conserved genes flanking a variable region comprising biosynthetic genes that encode glycosyltransferases. This variability is what underlies the diversity of EPS structures, as the glycosyltransferases are responsible for building the repeat units that are subsequently assembled into long polymers (Fig. 1). The repeat unit is synthesized on the inside of the plasma membrane, with the nascent glycoside anchored to the lipid membrane. The first step of the process is catalyzed by the priming glycosyltransferase, which primes the lipid anchor, usually undecaprenyl-phosphate, by attaching a sugar monomer to it (10). The priming glycosyltransferase is not a true glycosyltransferase, but a phosphoglycosyltransferase, and thus, the resulting product is undecaprenyldiphosphoglycoside. While EPS molecules can contain a large variety of sugar monomers, all LAB priming glycosyltransferases that have been characterized so far are either phosphoglucosyltransferases or phosphogalactosyltransferases.

After the first sugar monomer is attached, glycosyltransferases sequentially add the remaining sugar monomers until the entire repeat unit has been completed. The function of each glycosyltransferase can be described in terms of donor specificity, acceptor specificity, and mechanism of catalysis. Donor specificity determines *what* will be added, that is, which activated nucleotide sugar will provide the sugar monomer that is transferred (e.g., glucose, galactose, etc.). Acceptor specificity determines *where* the sugar will be added, that is, the substrate that can be glycosylated by the glycosyltransferase and thus the glycoside structure that must already be present in the nascent repeat unit for glycosylation to occur. Donor and acceptor specificities are usually determined by distinct domains in the glycosyltransferase protein; however, it has been shown that even small sequence variations in these domains can have substantial effects on the substrate specificity of the enzyme (11). Donor and acceptor specificities are thus difficult to predict from sequence. The mechanism of catalysis determines *how* the sugar will be added—most importantly, the anomeric configuration of the resulting glycoside. Glycosyltransferase mechanisms of catalysis can be divided into retaining and inverting mechanisms, indicating whether the anomeric configuration of the product is the same as or opposite to that of the donor (12). Since the nucleotide sugar donors for the Wzy-dependent pathway are always in the same anomeric configuration, for example, UDP-glucose always exists as UDP-α-glucose, the anomeric configuration of the product can be predicted from the mechanism of the glycosyltransferase. Although substrate specificity is difficult to predict from protein sequence, the mechanism of catalysis is generally conserved within CAZy families (13, 14) and is thus comparatively easy to predict from sequence. When the repeat unit has been completed, it is flipped to the outside of the cell membrane and added to an elongating chain of repeat units by a polymerase (10). It is worth noting that since the priming glycosyltransferase is a phosphoglycosyltransferase, the anomeric configuration of the priming sugar in the final polysaccharide is determined by the polymerase and not by the priming glycosyltransferase.

In many Wzy-dependent LAB EPS systems, repeat-unit assembly is expected to involve approximately one glycosyltransferase step per added residue, although putatively inactive genes and exceptions may occur. The final EPS structure primarily depends on the functions of these glycosyltransferases. With detailed functional characterization of all glycosyltransferases in an *eps* cluster, it would be possible to infer the resulting EPS structure. Although previous studies have successfully characterized some glycosyltransferases through heterologous expression and biochemical assays (15–20), the extensive work required and the vast diversity of *eps* glycosyltransferases limit the feasibility of this bottom-up approach. This study employs a computational top-down approach to putatively assign each glycosyltransferase to one or more monomers in the EPS repeat unit. These assignments are constrained by the predicted mechanism of catalysis and existing experimental results and leverage the partial overlap of *eps* genes and EPS structure among different LAB strains to link specific *eps* glycosyltransferases with particular structural features in the EPS.

## MATERIALS AND METHODS

### Strains, media, and culture conditions

*S. thermophilus* STCH_01, STCH_02, STCH_03, STCH_04, STCH_05, STCH_06, STCH_07, STCH_08, STCH_09, and STCH_10 are proprietary strains obtained from the Chr. Hansen Culture Collection. For polysaccharide isolation and purification, the strains were cultured in chemically defined medium (CDM) supplemented with 2% lactose (21). For inoculum preparation, bacteria were streaked from glycerol stock onto M17 (22) agar plates and incubated anaerobically overnight at 37°C. A single colony was inoculated into CDM and incubated anaerobically overnight at 40°C.

### Isolation and purification of exocellular polysaccharides

*S. thermophilus* strains were grown in CDM supplemented with 2% lactose under anaerobic conditions for 24 h at 40°C. The culture was then centrifuged (10,000 × *g*, 1 h, 4°C), and the supernatant was treated with 80% trichloroacetic acid (wt/vol) to a final concentration of 20%. The precipitation was allowed to proceed at 4°C with agitation for 2 h. The mixture was centrifuged (10,000 × *g*, 1 h, 4°C), and the crude EPS was precipitated by the addition of an equal volume of acetone (4°C) to the supernatant. After overnight precipitation at 4°C with mild agitation, the sample was centrifuged (10,000 × *g*, 1 h, 4°C). The pellet was washed three times with 50% acetone (4°C), and the resulting pellet was suspended in 50 mL distilled water with vigorous agitation. The suspension was subjected to sonication for 5 min, leading to a clear solution. Finally, this solution was dialyzed for 12 h against distilled water (5 L) at 4°C. The dialysis was repeated two more times, each time against fresh distilled water. The content of the dialysis bag was freeze-dried, and the EPS residue was solubilized in 1 mL D_2_O by vigorous agitation and sonication.

### Monosaccharide analysis

The EPS (1.5 mL) was mixed with 660 µL of trifluoroacetic acid at 13 M (final concentration = 4 M) in a glass inoculum bottle (10 mL), and the bottle was closed with a rubber cap. The mixture was degassed with pure argon for 20 min and incubated for 5 h at 80°C. After hydrolysis, the trifluoroacetic acid was removed by evaporation at negative pressure in a rotavapor at 40°C. The remaining solution was washed three times with 4 mL of methanol. The methanol was evaporated between each washing step. Hydrolyzed samples were freeze-dried overnight, and the powder was dissolved in 600 µL D_2_O and analyzed by thin-layer chromatography (TLC) and ^1^H-NMR to determine the monosaccharide composition.

### Structural elucidation of EPS by NMR

The spectra were acquired on an AVANCE III 800 spectrometer (Bruker, Rheinstetten, Germany) working at a proton operating frequency of 800.33 MHz, equipped with a three-channel inverse detection probe (TXI) with pulse-field gradients. Two-dimensional spectra for structural elucidation were acquired at 60°C using standard Bruker pulse programs. Proton-homonuclear shift correlation spectroscopy (COSY), total correlation spectroscopy (TOCSY), ^1^H-^13^C multiplicity-edited heteronuclear single quantum coherence spectra (ed-HSQC), and two-dimensional HSQC-TOCSY were used for the complete assignment of all the resonances in the HSQC maps. ^3^*J*_H1,H2_ as measured directly from the 1D ^1^H spectra and ^1^*J*_C,H1_ as measured from non-decoupled HSQC spectra were used to establish the configurations of the sugar rings. Heteronuclear multiple bond connectivity spectra (HMBC) were acquired to determine the position of glycosidic bonds. The identity of the sugar monomers was established by ^1^H and ^13^C chemical shift patterns and, when possible, by the determination of distinct ^3^*J*_H,H_ couplings. Descriptions of the interpretation of NMR spectra for each isolated polysaccharide can be found in the supplemental material.

### Identification of *eps* gene clusters

If an entry in NCBI contained the *eps* cluster for a given strain, this record was used. In cases where only a whole genome sequence was available, the *eps* cluster was extracted. As *eps* clusters have conserved genes at both ends, they were identified using BLAST by searching translated gene sequences of the annotated genome for the known protein sequences of the first and last gene of the cluster. The organization of genes within the *eps* clusters in LAB, as reviewed by Zeidan et al. (1), was used to determine the first and last *eps* genes in each individual species, for example, *epsR* and *orfY* in *Lactococcus lactis* and *epsA* and *orf14.9* in *S. thermophilus*. If a single hit with over 90% sequence identity and over 90% coverage was found for both the first and the last genes, all genes between and including the two hits were extracted and used as the *eps* cluster. NCBI accession numbers of all the used *eps* clusters can be found in the supplemental material.

### Prediction of the glycosyltransferase catalysis mechanism

Glycosyltransferase families were predicted using the tools CUPP (23) and dbCAN-HMM (24). These prediction tools are both based on the CAZy database of carbohydrate-active enzymes (13) but use complementary methods: k-mer patterns and profile hidden Markov models, respectively. If one tool made several predictions on a single sequence, the prediction with the highest score was selected. To avoid erroneous family assignment, only predictions made concordantly by the two tools were used. Based on the CAZy family predictions, the predicted mechanism of catalysis (inverting or retaining) was assigned according to CAZy annotations.

### Comparisons of glycosyltransferases

Sequence similarity was assessed by pairwise global alignment of amino acid sequences, using the BLOSUM62 scoring matrix, a gap opening penalty of 10, and a gap extension penalty of 1. For priming glycosyltransferases, a distance matrix of 100 – percent_identity was constructed, and agglomerative hierarchical clustering (complete linkage) was used to create a dendrogram of the sequences.

For non-priming glycosyltransferases, all pairs were aligned to each other, and the percent identity was calculated. The pairwise identities showed a strong bimodal distribution, with almost all pairs having <55% or >95% identity. All pairs of glycosyltransferases with more than 90% sequence identity were identified and used in the last step of the functional assignment described below.

### Functional assignment of glycosyltransferases

An optimization procedure was developed with the goal of putatively assigning each glycosyltransferase to the monomer(s) in the EPS structure that it can possibly add. The procedure consisted of several phases, where assignments were iteratively narrowed down, using different rules and sources of evidence.

- Phase 0: Initial assignment of individual glycosyltransferases to monomers.
- Phase 1: Joint assignment of glycosyltransferases in each strain to find combinations where each monomer is ideally assigned exactly one glycosyltransferase.
- Phase 2: Integration of experimental evidence to keep only solutions that are consistent with experimentally validated glycosyltransferase functions.
- Phase 3: Utilization of glycosyltransferase sequence identity between strains to keep only solutions where pairs of highly similar glycosyltransferases are assigned to the same linkage triplet.

In Phase 0, the possible assignments of each individual glycosyltransferase were constrained by the following rules: Priming glycosyltransferases could only be assigned to glucose and galactose monomers in the main chain of the structure, and non-priming glycosyltransferases could only be assigned to monomers with an anomeric configuration consistent with the mechanism of catalysis associated with the predicted CAZy family.

In Phase 1, each strain was optimized separately, with the goal of finding combinations of assignments for all of the strain’s glycosyltransferases that minimize redundancies or incompleteness, that is, zero or two or more glycosyltransferases being assigned to the same monomer. From the Phase 0 assignments, combinatorial optimization was used to find the best combinations of assignments by enumerating all possible combinations of the initial assignments, and scoring each possible solution using an objective function:


$$f=\sum_{i\in M}|k_{i}-1|$$

where *M* is the set of monomers in the strain’s repeat unit, and *k_i_* is the number of glycosyltransferases assigned to monomer *i*. All solutions with the minimal possible objective score were kept.

In Phase 2, the number of solutions for each strain was further reduced by integrating known functional annotations. This included inferred substrate specificity of priming glycosyltransferases as well as the results of experimental characterizations of glycosyltransferases found in the literature (15–20). Solutions from Phase 1 that were not consistent with these known functional annotations (i.e., a glycosyltransferase being assigned to a monomer other than the one shown experimentally) were discarded.

The final step, Phase 3, used sequence identity between strains to further reduce the set of possible solutions. Pairwise sequence identities for all glycosyltransferases were calculated using global pairwise alignment. All pairs of strains that shared at least one pair of glycosyltransferases with over 90% sequence identity were analyzed individually. A set of potential solutions for the pair of strains A and B was generated by the Cartesian product of preliminary solutions for strain A and preliminary solutions for strain B. As a working assumption in the optimization framework, glycosyltransferases with over 90% sequence identity were considered likely to have identical or highly similar substrate specificities, and thus, the objective function that was used minimized the number of pairs of similar glycosyltransferases that were not assigned to identical linkage triplets [e.g., *alpha-Glc-(1–4)-beta-Gal*]. All strain-pair solutions with the minimal objective score were kept. The final set of solutions for each strain was generated by the intersection of solutions from all strain pairs it participated in. The possible assignments for a given glycosyltransferase were then determined from the set of assignments in all the remaining solutions.

## RESULTS

### Identification of EPS structures and *eps* clusters

A total of 66 distinct EPS structures were found to have been published so far (1, 25, 26). For 15 strains, corresponding *eps* gene cluster sequence data could be retrieved from the NCBI, and we additionally elucidated the EPS structures of 10 *S. thermophilus* strains from the Chr. Hansen Culture Collection by NMR spectroscopy. These 10 strains comprised nine distinct EPS repeat unit structures, four of which were not previously reported in the literature. The structural assignments are supported with different levels of confidence across the data set; for some strains, the repeat unit could be assigned with high confidence based on well-resolved COSY, TOCSY, HSQC, HSQC-TOCSY, and HMBC correlations and/or direct agreement with previously reported structures, whereas for others, the assignments rely more heavily on integrated interpretation of partially overlapping spectra, limited long-range correlations, or comparison with related EPS structures. Accordingly, the supplemental material provides strain-specific descriptions of the spectral evidence and the main limitations encountered for each structure. NCBI accession numbers for the included sequences, the elucidated repeat unit structures, as well as NMR spectra and detailed descriptions of their interpretation can be found in the supplemental material. The 25 repeat unit structures, for which corresponding gene cluster sequences were available, encompassed a total of 142 monomers. The corresponding 25 *eps* clusters contained a total of 144 glycosyltransferase genes.

### Priming glycosyltransferases

**Fig 2:**
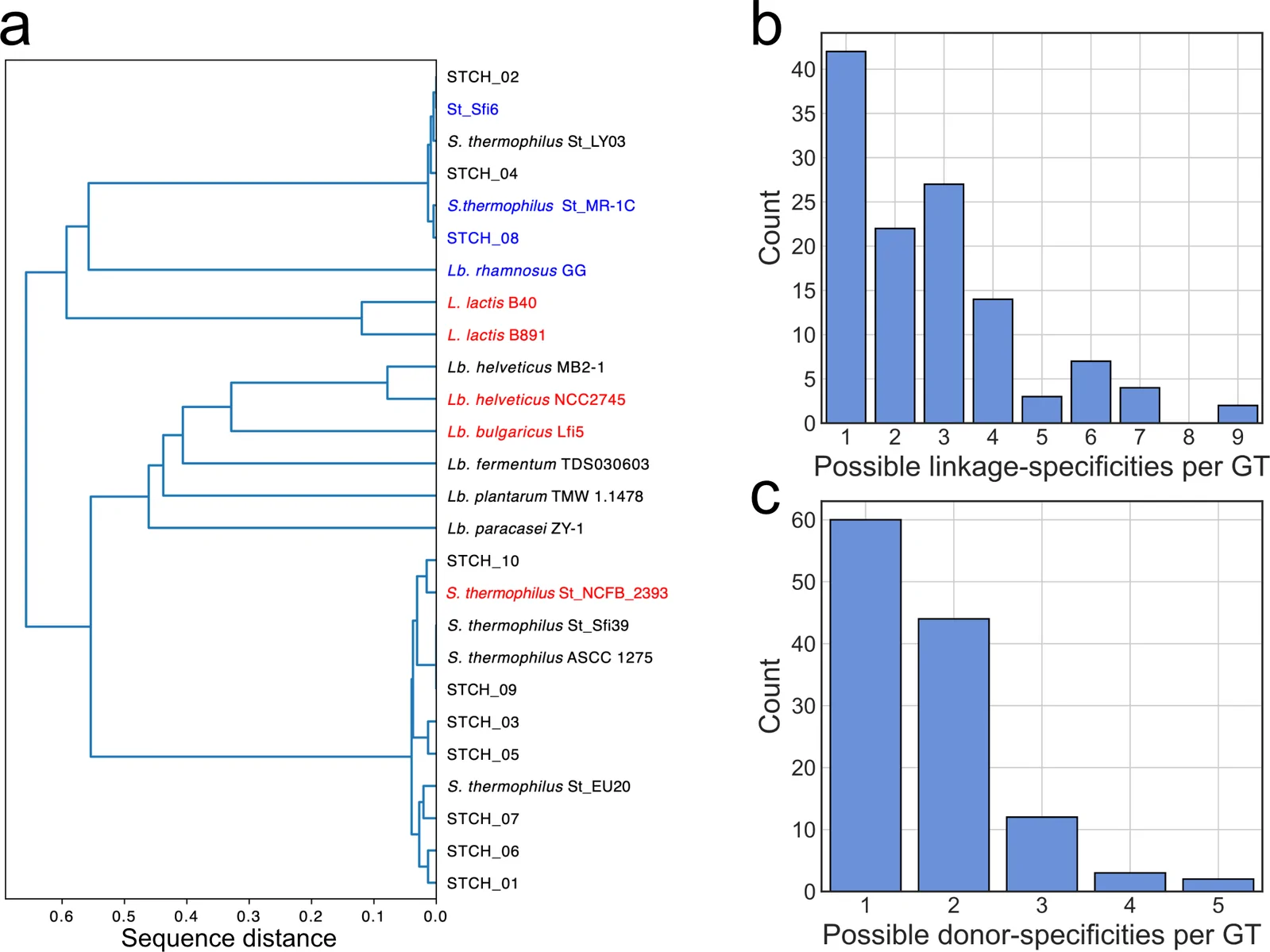
Overview of glycosyltransferase specificities. (**a**) Dendrogram based on pairwise protein sequence identities between the priming glycosyltransferase from each strain. Strains written in blue have a priming glycosyltransferase that is known experimentally or through inference to be galactose-specific. Strains written in red have a priming glycosyltransferase that is known experimentally to be glucose-specific. (**b**) Histogram showing the distribution of the number of possible linkage specificities as determined by combinatorial optimization. (**c**) Histogram showing the distribution of the number of possible donor-sugar specificities as determined by combinatorial optimization. Panels b and c include only glycosyltransferases for which “Inactive” was not a possible solution.

A comparative analysis was performed for the protein sequences of the priming glycosyltransferases identified in the *eps* clusters. Although all the sequences contain the same Pfam domain, PF02397.16 (14), significant diversity was observed, with the lowest pairwise sequence identity between two sequences being 34%. Two distinct clusters containing sequences with over 95% sequence identity to each other were observed among the priming glycosyltransferase sequences from *S. thermophilus*. The substrate specificities of some of these proteins have previously been characterized, and some can be inferred from a lack of glucose in the main chain of the corresponding EPS structure. The known and inferred substrate specificities separate between the two clusters, as shown in Fig. 2a, which indicates, together with the very high sequence identity within each cluster, that one cluster contains phosphoglucosyltransferases while the other contains phosphogalactosyltransferases. For the remaining priming glycosyltransferases whose function has not been previously characterized, no functional inference could be made, except that they were all assumed to transfer either glucose-1-phosphate or galactose-1-phosphate.

### Functional assignment of glycosyltransferases

All *eps* glycosyltransferases were analyzed with the CUPP pipeline (23) and dbCAN-HMM (24) to predict their respective CAZy families. Of the 119 non-priming glycosyltransferases, 75 could be confidently assigned to a family, based on concordant predictions by the two tools. The most commonly predicted families were GT2 (16) and GT4 (27), while GT14 (4), GT32 (4), GT11 (1), and GT101 (1) were also predicted. Consequently, 38 glycosyltransferases were predicted to be inverting, while another 36 were predicted to be retaining. The sequence predicted as family GT101 could not be assigned a mechanism, as the mechanism for GT101 is currently not known.

Running the procedure for functional assignment based on the combinatorial optimization approach described in the Materials and Methods resulted in a list of monomers that each glycosyltransferase could possibly add.

Table 1 shows an overview of the glycosyltransferases that can possibly add each of the monomers in each EPS structure. Of the total 144 glycosyltransferases, 42 could be uniquely assigned to a single monomer in the repeat unit, while 14 were uniquely determined to be putatively inactive. Of the glycosyltransferases that were found to be active but could not be uniquely assigned to a monomer, 22 glycosyltransferases were assigned to two possible monomers, and 27 glycosyltransferases were assigned to three possible monomers (Fig. 2b). Besides the 42 glycosyltransferases that were uniquely assigned to a monomer, the sugar donor specificities of 18 additional glycosyltransferases could be uniquely determined because all the assigned monomers were of the same sugar type. Thus, the sugar donor specificities of a total of 60 glycosyltransferases could be uniquely determined Fig. 2c.

**TABLE 1:** EPS repeat unit structures for 25 strains of LAB^*a*^

Strain	Structure	Structure source
*S. thermophilus* STCH_06	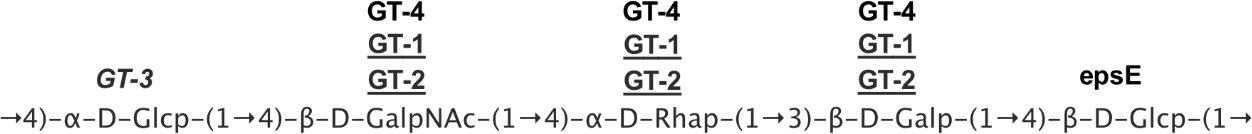	This work
*S. thermophilus* STCH_08	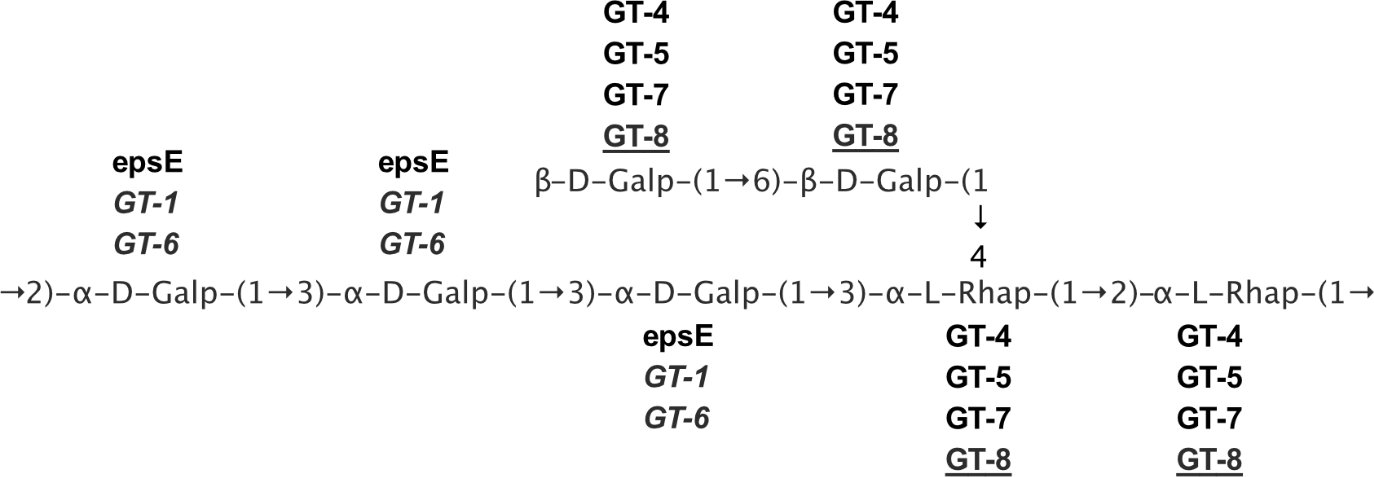	This work
*S. thermophilus* STCH_01	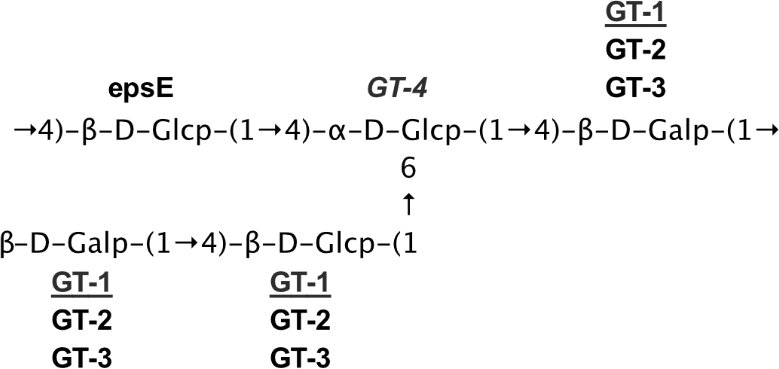	This work
*S. thermophilus* STCH_05	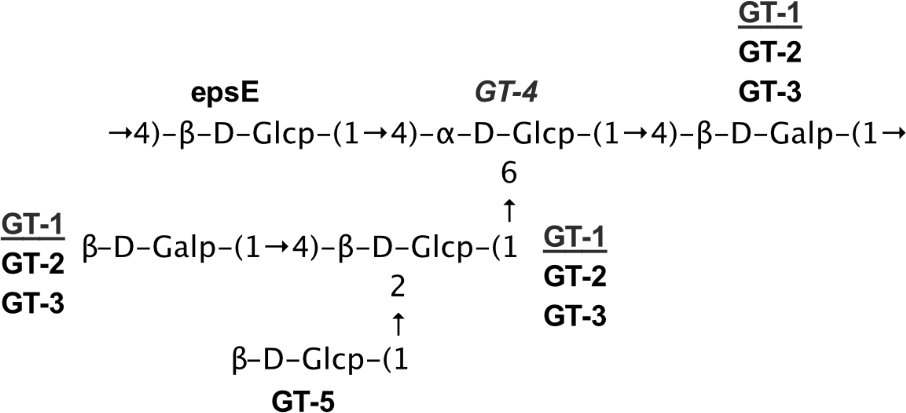	This work
*S. thermophilus* STCH_07	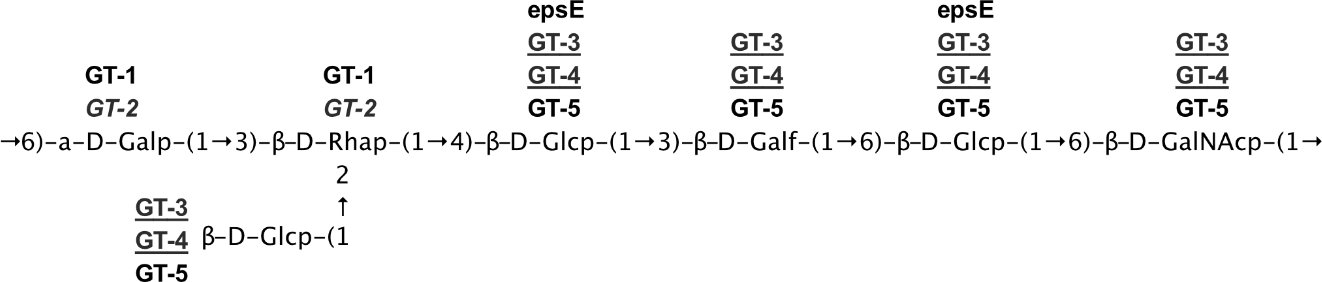	This work
*S. thermophilus* STCH_10	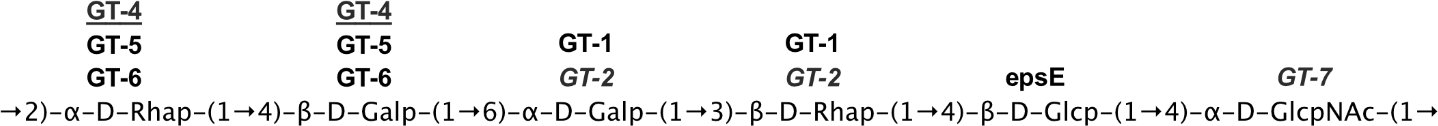	This work
*S. thermophilus* STCH_03	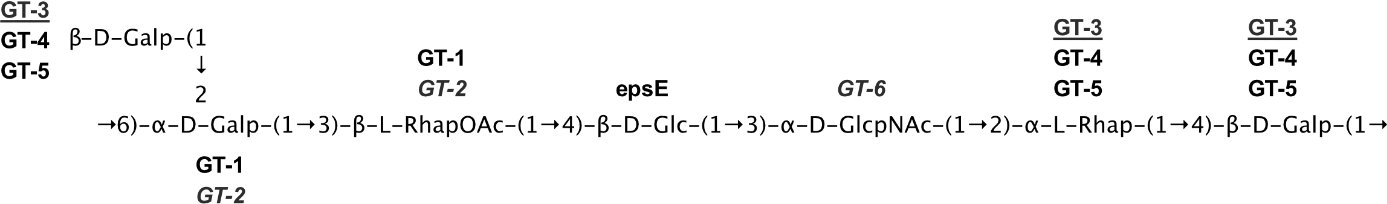	This work
*S. thermophilus* STCH_04	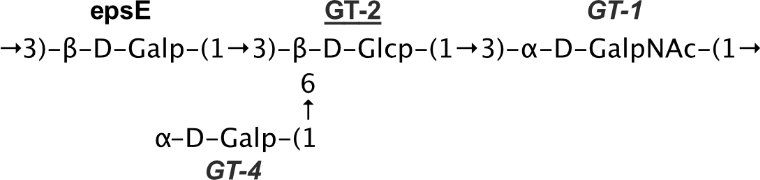	This work
*S. thermophilus* STCH_02	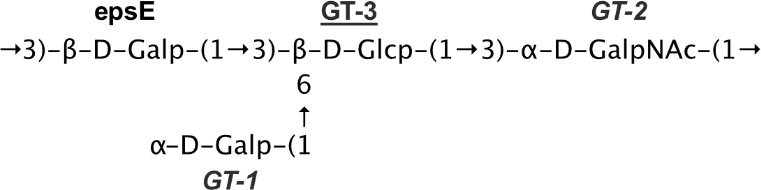	This work
*S. thermophilus* Sfi6	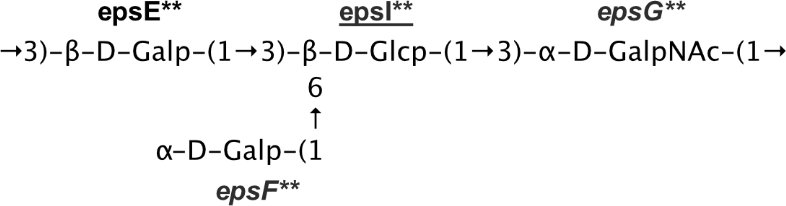	(28)
*S. thermophilus* LY03	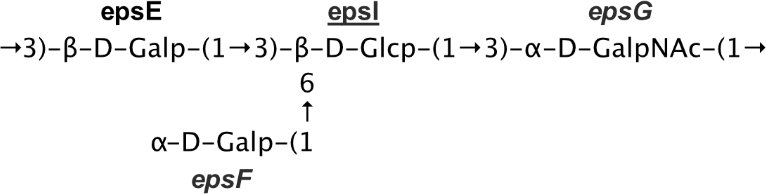	(29)
*S. thermophilus* STCH_09	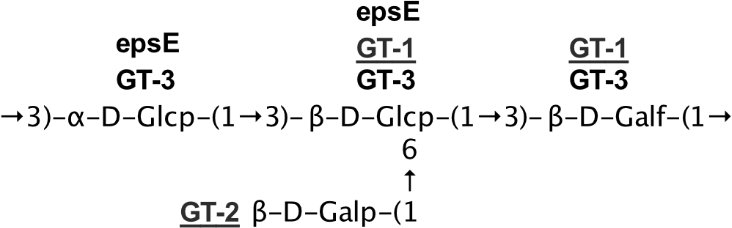	This work
*S. thermophilus* Sfi39	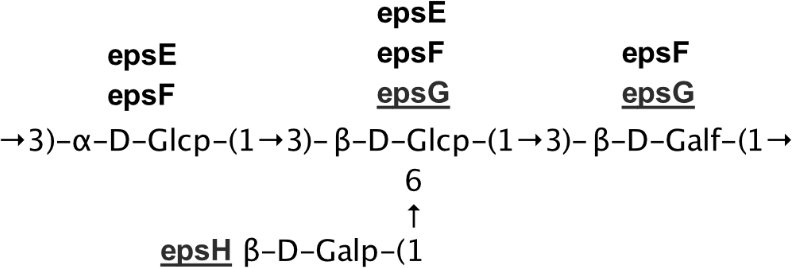	(30)
*S. thermophilus* ASCC 1275	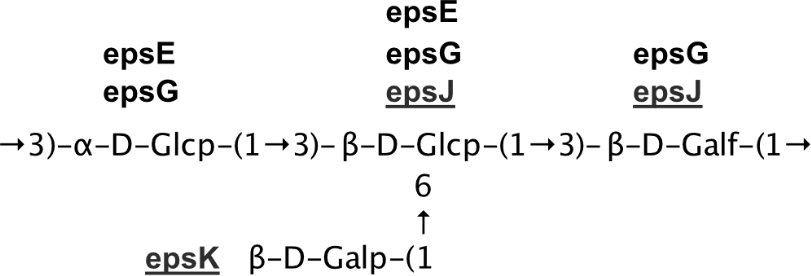	(31)
*L. lactis* B40	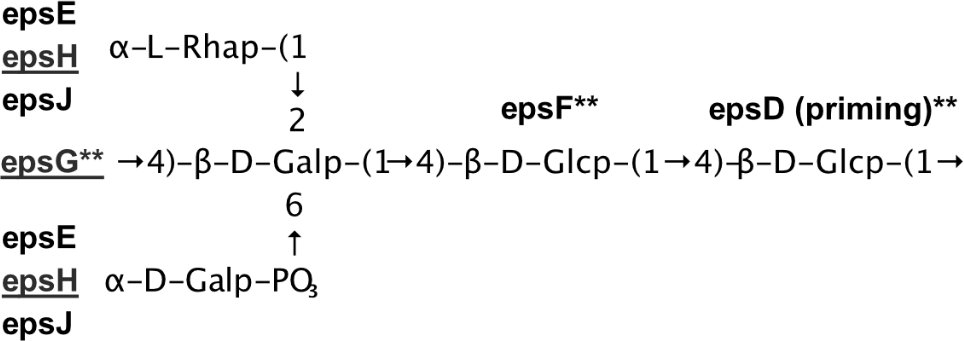	(32)
*L. lactis* B891	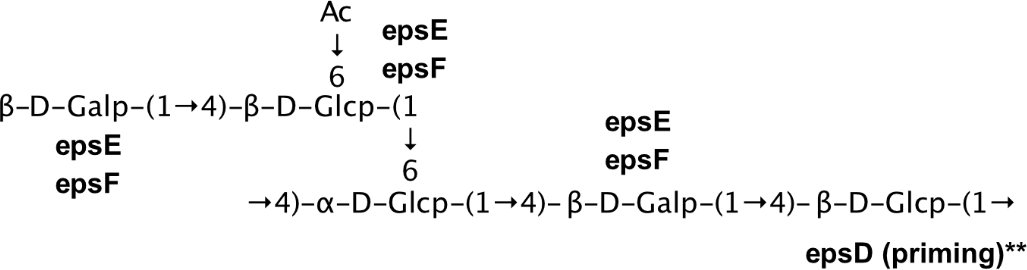	(27)
*Lb. delbueckii subsp. bulgaricus* Lfi5	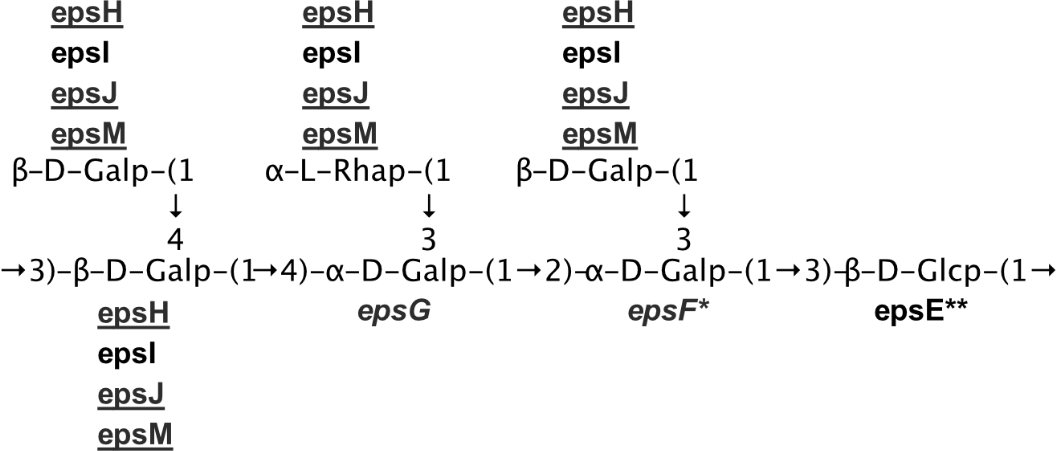	(16)
*Lb. fermentum* TDS030603	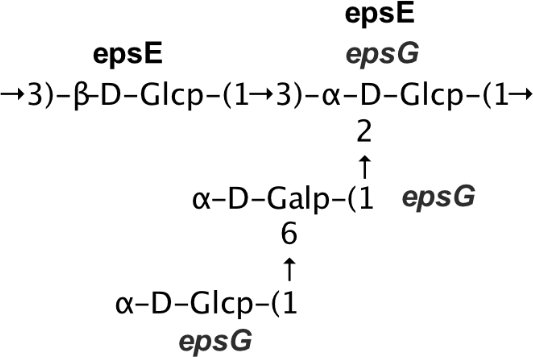	(33)
*Lb. helveticus* NCC2745	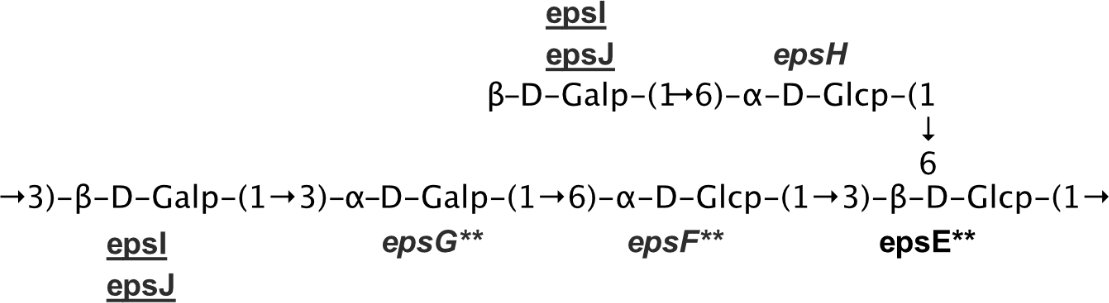	(20)
*Lb. rhamnosus* GG	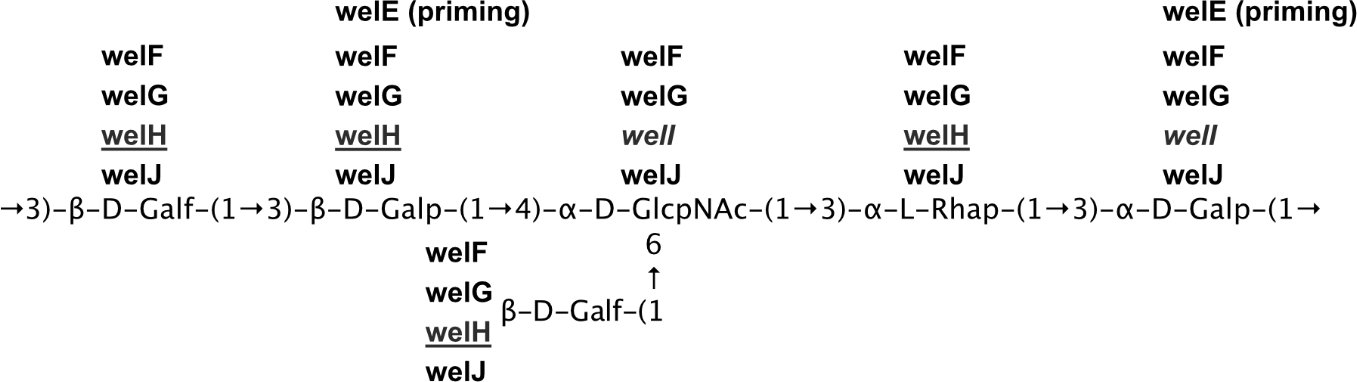	(34)
*Lb. plantarum* TMW 1.1478	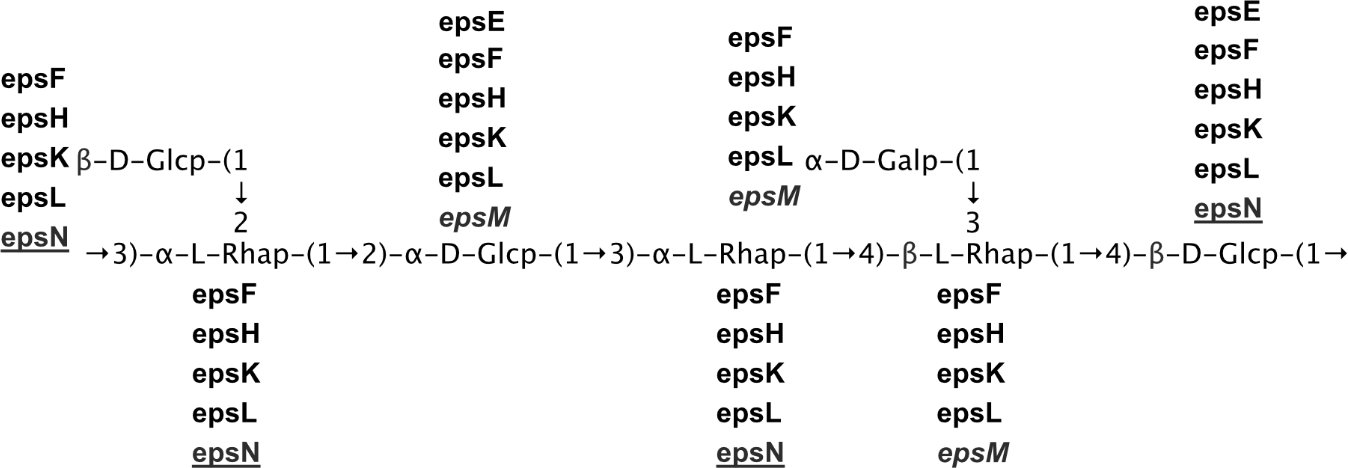	(35)
*Lb. paracasei* ZY-1	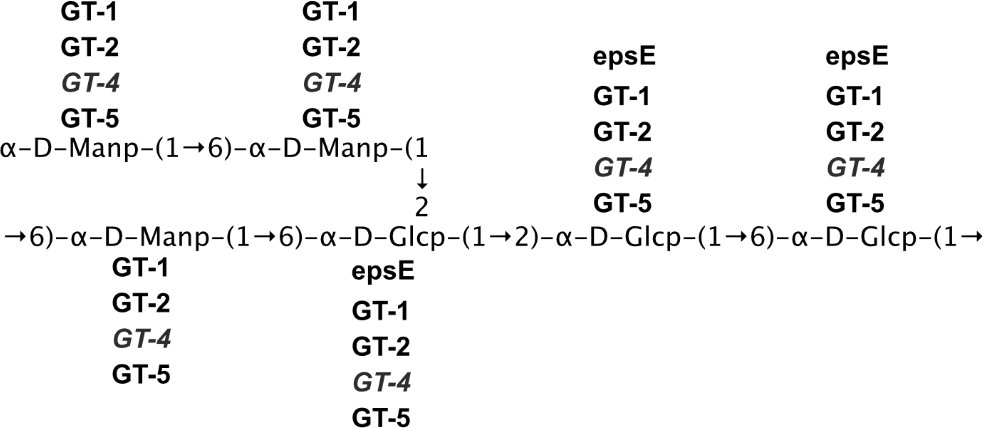	(36)
*Lb. helveticus* MB2-1	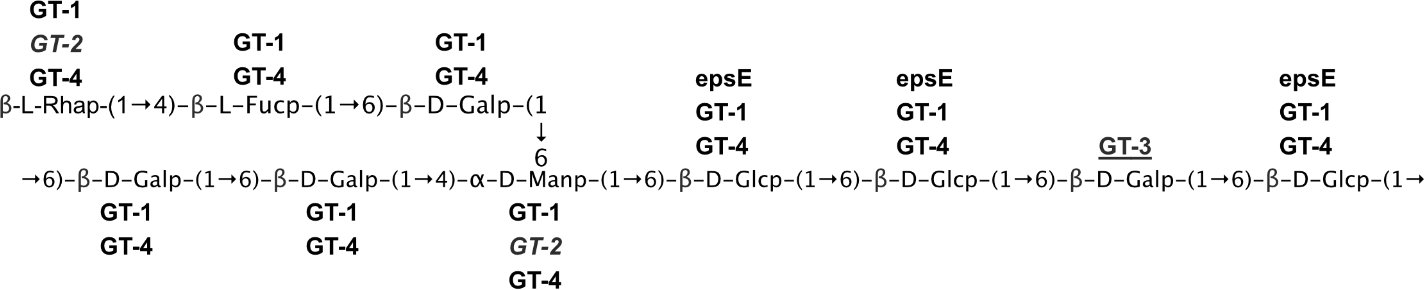	(37)
*S. thermophilus* EU20	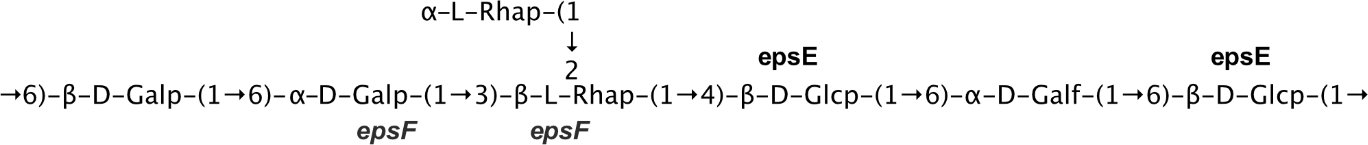	(38)
*S. thermophilus* MR-1C	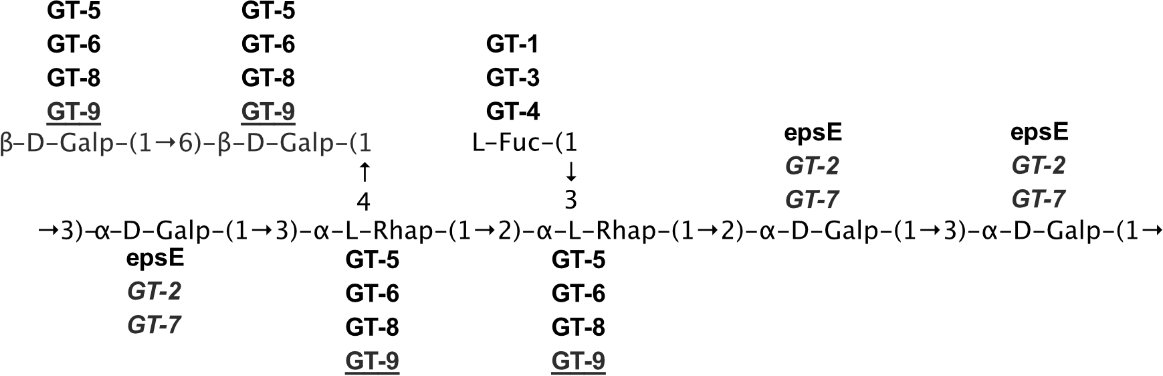	(4)

^*a*^ The results of the functional assignment of glycosyltransferases are indicated by the glycosyltransferases listed next to each monomer. Glycosyltransferases with underlined names are predicted to be inverting, while glycosyltransferases in italics are predicted to be retaining. The remaining glycosyltransferases are either priming phosphoglycosyltransferases or could not be confidently assigned a mechanism. Unless otherwise specified, epsE is used to denote the priming glycosyltransferases. ** indicates functional assignments based on experimental observations obtained from the literature.

## DISCUSSION

For some strains, such as *Lb. rhamnosus* GG and STCH_06, only very little new information could be gained, and consequently, very few, if any, unique assignments could be made. This group contained strains that did not share glycosyltransferases with more than 90% sequence identity with any other strain, and where the only source of information was thus from predicted mechanisms of catalysis. For another group of strains, unique assignments could be made based on similarity with strains where gene-structure associations have been experimentally established. For instance, the strains STCH_04 and STCH_02 were found to have EPS structures identical to that of *S. thermophilus* Sfi6, where all glycosyltransferases have been experimentally characterized (17). As the *eps* clusters of these three strains were also virtually identical, the glycosyltransferases of strains STCH_04 and STCH_02 could be trivially assigned to monomers corresponding to the experimental assignments in *S. thermophilus* Sfi6.

The strains from which the most information could be extracted in this analysis were those that showed partial similarities in EPS structures and *eps* clusters with other strains. For example, the strains STCH_05 and STCH_01 share five monomers in their EPS structures, with STCH_05 having an extra branching beta-glucose. As STCH_05 only contained one glycosyltransferase that was not found in STCH_01, this protein could be uniquely assigned to the branching beta-glucose.

Similarly, the three strains STCH_10, STCH_07, and STCH_03 contain the trisaccharide α*-Gal-(1–3)-ß-Rha-(1–4)-ß-Glc*, of which the glucose was found to be the priming sugar. These strains share the priming glycosyltransferase and two additional glycosyltransferases, which are thus likely to add the three sugars in the trisaccharide. Additionally, one of the shared glycosyltransferases has 78% sequence identity with a *Streptococcus pneumoniae* glycosyltransferase, which has previously been found to form a *ß-Rha-(1–4)-ß-Glc* linkage (39). Although sequence similarity to *S. pneumoniae* was not included in the automatic assignment pipeline, this putative rhamnosyltransferase likely adds a beta-rhamnose to the priming glucose, while the other shared glycosyltransferase then adds the alpha-galactose. The same three glycosyltransferases are also found in the strain *S. thermophilus* NCFB2393, whose EPS repeat unit structure, to our knowledge, has not been experimentally determined. We thus hypothesize that *S. thermophilus* NCFB2393 also contains the *α-Gal-(1–3)-ß-Rha-(1–4)-ß-Glc* trisaccharide.

Each glycosyltransferase’s mechanism of catalysis was predicted based on the CAZy family and used to constrain possible assignments to the linkages matching the mechanism. This significantly reduces the search space as glycosyltransferases that could not be confidently placed in a CAZy family would have roughly twice as many possible linkage assignments as those that could be assigned a CAZy family. Since a substantial minority (44/119) of the glycosyltransferases could not be assigned a CAZy family, the number of unique final linkage assignments could likely be increased by improving the CAZy family predictions.

The issue of associating polysaccharide glycosyltransferases with individual chemical features of the polysaccharide structure has previously been addressed in *S. pneumoniae*. By correlating the presence of glycosyltransferase homology groups to the presence of specific linkages (39), the authors putatively identified the glycosyltransferases responsible for 145 out of 200 capsular polysaccharide linkages present in 88 different repeat unit structures in *S. pneumoniae*. The success of such a relatively simple approach compared to the optimization approach used in the present study might, in part, be explained by the larger number of available repeat unit structures in *S. pneumoniae*. However, in addition to the difference in data quantity, an important difference between the two data sets is the degree of variability. In the *S. pneumoniae* data, many repeat unit structures differed from each other in only a single linkage (14), whereas in *S. thermophilus*, such cases, as previously mentioned, were rare, making the association between individual glycosyltransferases and linkages more ambiguous. The pneumococcal capsular polysaccharide is known to be an important factor in the interaction with host immune responses, and a selective pressure for immune system evasion might thus have created high diversity in the biosynthetic cluster of *S. pneumoniae* (14). A similar selective pressure is unlikely to have affected the recent evolution of *S. thermophilus*, which might thus have more conserved *eps* clusters within strain families.

While this conservation can facilitate comparative assignment of glycosyltransferase function, it also underscores that the current framework depends on the nature and extent of the available data. The analysis therefore has several limitations. The number of strains with both a well-resolved EPS repeat unit structure and a corresponding *eps* cluster sequence is still limited, reducing the amount of comparative information available for assignment. In addition, the framework depends on CAZy family-based predictions of glycosyltransferase catalytic mechanism and on the assumption that proteins sharing more than 90% sequence identity have identical specificities. Consequently, the most certain assignments (i.e., assignment to fewer possible linkages) are made for glycosyltransferases with a CAZy family prediction and with close homologs in other strains. However, even high-certainty assignments may, in rare cases, be incorrect, for example, due to erroneous CAZy family predictions or highly similar proteins where small sequence differences cause a change in specificity. Despite these limitations, the assignments, even those only partially resolved, can be used in practice to generate testable hypotheses, narrow down potential gene functions, and guide targeted experimental validation of specific glycosyltransferases or linkages. As more paired structural and genomic data become available, this framework should become increasingly powerful for resolving glycosyltransferase function and advancing genome-based prediction of EPS structure in LAB.

### Summary

We elucidated the repeat unit structure of EPS from 10 strains of *S. thermophilus*, which revealed four novel repeat unit structures not previously observed in LAB. By employing a computational approach that integrates comparative analyses of *eps* clusters and EPS structures, predictions of glycosyltransferase catalytic mechanisms, and experimental evidence, we have putatively assigned *eps* cluster glycosyltransferases to specific monomers in the corresponding EPS repeat units. This work provides a scalable framework for mapping EPS gene-structure relationships and for improving genome-based prediction of LAB polysaccharide repeat-unit composition.

### Conclusions

LAB produce EPS that are important for both food functionality and health-related applications. Here, we present a systematic computational framework for linking *eps* cluster glycosyltransferases to EPS repeat-unit structure by combining sequence comparison, predicted catalytic mechanism, experimental evidence, and comparative analysis across strains. Using this approach, we generated putative specificity annotations for a substantial fraction of the glycosyltransferases analyzed across several lactic acid bacterial species. While further progress will depend on the availability of additional matched structural and genomic data sets, this study advances the mapping of EPS genotype-phenotype relationships in LAB. This knowledge can improve genome-guided strain screening, support starter culture selection, and contribute to textural optimization in dairy and plant-based fermented products. Because the framework is systematic and extensible, it can be readily updated as new data emerge.

## SUPPLEMENTAL MATERIAL

Supplemental material aem.00612-26-s0001.docx EPS structure elucidation, data, and code.

ASM does not own the copyrights to Supplemental Material that may be linked to, or accessed through, an article. The authors have granted ASM a non-exclusive, world-wide license to publish the Supplemental Material files. Please contact the corresponding author directly for reuse.
