# Supplementary material for: Linking exocellular polysaccharide structures and biosynthetic genes in lactic acid bacteria by combinatorial optimization
Source: Appl Environ Microbiol. 2026 Jul 20;92(8):e00612-26. doi: 10.1128/aem.00612-26 (PMC13488360; doi:10.1128/aem.00612-26)
Supplement: Supplementary file 1 [file aem.00612-26-s0001.docx]

# **Supplementary Methods: Structural elucidation of EPS by NMR**

**STCH_04 EPS structure**

Preliminary analysis of the ^1^H spectrum revealed an EPS with a repeating unit composed of 4 saccharide residues (4 signals in the anomeric region of the spectrum). The presence of a signal at 2.33 (a singlet) was indicative of an acetylation.

EPS was completely hydrolysed to its saccharide units and the monomers tentatively assigned by thin layer chromatography. ^1^H-NMR analysis of this sample revealed the presence of galactose, glucose, and galactosamine (in small amount due to the probable hydroxylation into galactose by the hydrolysis procedure). The identification of the monomeric units by NMR is consistent with the results obtained from TLC.

The EPS of STCH_04 has only four residues in the repeating unit, so, in principle, the assignment of its ^13^C/^1^H HSQC spectrum signals should be relatively straight forward. However, it was never possible to produce a sample with enough concentration to acquire a good quality HSQC-TOCSY or HMBC spectra (the first one being extremely helpful in the assignment process and the other in the establishing of the position of the glycosidic bonds). Nevertheless, with the use of COSY, TOCSY and ^13^C-HSQC spectra, and comparison with ^13^C chemical shift values from the literature, an integrated analysis of the data allowed the complete and reasonably reliable assignment of all the resonances (Table S1) (Figs S1).

Table S1 – Assignment of the HSQC signals of the repeating unit of STCH_04 EPS.

| Unit/position | 1 | 2 | 3 | 4 | 5 | 6 |
| --- | --- | --- | --- | --- | --- | --- |
| A ^1^H (ppm) | 5.39 | 4.71 | 4.45 | 4.30* | 4.21* | nd |
| A ^13^C (ppm) | 94.66 | 48.71 | 77.90 | 69.69* | 71.33* | 61.49 |
| B ^1^H (ppm) | 5.29 | 4.15 | 4.15 | 4.52* | 4.51* | nd |
| B ^13^C (ppm) | 98.64 | 70.04 | 68.87 | 69.10* | 71.10* | 61.49 |
| C ^1^H (ppm) | 5.01 | 4.01 | 4.08 | 4.41 | 3.98 | nd |
| C ^13^C (ppm) | 103.68 | 70.28 | 77.19 | 65.35 | 75.55 | 61.49 |
| D ^1^H (ppm) | 4.91 | 3.82 | 4.06 | 3.91 | 3.95 | 4.24 ; 4.08 |
| D ^13^C (ppm) | 104.27 | 73.09 | 85.05 | 68.64 | 74.50 | 66.41 |

nd- not determined; the position 6 of residues A,B, and C are all overlapped at 61.49 ppm in ^13^C and between 4.08 and 3.97 ppm in ^1^H. *The values for position A4 and B4 may be switched; the same is true for positions A5 and B5.

The acetyl group at position 2 of the galactoseamine presents a methyl group resonating at 22.57 ^13^C ppm and 2.32 ^1^H ppm with its carboxyl group generating a signal at 174.94 ^13^C ppm.

Analysis of the chemical shift values and coupling constants (Table S2) allowed us to conclude that: A is a 2-*N*-acetyl-galactoseamine in the alpha pyranosyl configuration; B is a galactose unit in the alpha pyranosyl configuration; C is a galactose in the beta pyranosyl configuration; and D is a glucose unit in the beta pyranosyl configuration.

Table S2 – Coupling constants of position 1 of the repeating unit of STCH_04 EPS.

| Unit | A | B | C | D |
| --- | --- | --- | --- | --- |
| ^1^J_CH1_ (Hz) | 177 | 177 | 168 | 163 |
| ^3^J_H1H2_ (Hz) | 3.3 | 2.5 | 7.1 | 7.9 |

*A value of ^1^J_CH_ around or above 170Hz is representative of an alpha pyranose configuration while a value between 160 and 170 is typical of the beta configuration. A value of ^3^J_H1H2_ around 3-4Hz is related to an alpha configuration, a value around 7-8 a beta configuration and a value below 2Hz is a consequence of a proton at an equatorial orientation at position 2 (the case of rhamnose or mannose).*

Since the HMBC spectrum did not present the required intensity for glycosidic bond position determination, the NOE effect was used. In the NOESY spectrum it was possible to detect the following contacts: A1-C3, B1-D6, C1-D3 and D1-A3. So, the repeating unit is (Fig. S2):

This is the same structure found by Stingele et al (1996), confirmed by Lemoine (personal communication to the authors of Stingele et al) and the same as the one found by Doco et al. (1990).

Figure S1 – ^13^C/^1^H HSQC spectrum of STCH_04 EPS (A) and respective expansion of the central region (B).

Figure S2 - Schematic representation of the structure of the repeating unit of STCH_04 EPS

**STCH_02 EPS structure**

A comparison of the HSQC spectrum of STCH_02 EPS with that of a sample containing STCH_04 EPS revealed the presence of the same repeating unit (Figs.S3 and S4).

Figure S3 – ^13^C/^1^H HSQC spectrum of: A- STCH_02 EPS and B- STCH_04 EPS

Figure S4 – Superimposition of HSQC spectra of STCH_02 (red) and B- STCH_04 (blue) EPSs.

So, the repeating unit of STCH_02 is (Fig. S5)

→3)-β-D-Gal*p*-(1→3)-β-D-Glc*p*-(1→3)-α-D-Gal*p*2*N*Ac-(1→

6

↑

α-D-Gal*p*-(1

Figure S5 - Schematic representation of the repeating unit of STCH_02 EPS.

**STCH_03 EPS structure**

Preliminary analysis of the ^1^H spectrum of STCH_03 EPS revealed the presence of 8 signals in the anomeric region (one of which does not correspond to an anomeric proton (see below)), two methyl singlets in the region where acetyl groups generally resonate (2.51 and 2.33 ppm), and two methyl doublets (at 1.66 and 1.61 ppm) indicative of two rhamnose residues.

STCH_03 EPS was hydrolysed to its saccharide units and the hydrolysate analysed by ^1^H-NMR. The hydrolysate revealed the presence of galactose, glucose, rhamnose and glucosamine (in small amount due to the probable partial hydroxylation into glucose by the hydrolysis procedure). The relative quantities of saccharide units in the original EPS were not possible to measure accurately because of the possible conversion of glucosamine into glucose and because the hydrolysis was not complete (several minor polisacharides were still present). Nevertheless, galactose was the main component followed by rhamnose, glucose and glucosamine, in this order.

The analysis of the ^13^C/^1^H HSQC spectrum (Fig. S6) allowed us to take the following conclusions: 1) the repeating unit of STCH_03 is composed of seven residues (there are only seven signals in the anomeric region in opposition to the apparent eight observed in the proton spectrum, the eighth signal is the consequence of an O-acetylation, see below); 2) the two ^1^H methyl singlets correlate to ^13^C signals around 20-22 ppm, indicative of two acetylations, and; 3) the two ^1^H methyl doublets correlate to ^13^C signals around 17 ppm, indicative of two methyls at position 6 of an hexose, considering that there are two anomeric signals with ^3^J_HH_ values below 2 Hz and that rhamnose was detected in the hydrolysate, we are in the presence of two rhamnose residues.

The fact that STCH_03 has a heptasaccharide as a repeating unit led to a considerably difficult spectral interpretation due to severe overlap, nevertheless, with the combined use of COSY, TOCSY, ^13^C-HSQC, ^13^C-HSQC-TOCSY and also using information from ^13^C-HMBC spectra, it was possible to achieve a complete and reasonably reliable assignment of all the resonances (Table S3) (Fig. S6).

The acetylations occur at positions 2 of residues C and D, as could be proven by correlations in the ^13^C-HMBC spectrum. In the case of D2 the carbon signal (53.90 ppm) is in the expected range for an aminated carbon, so we are in the presence of an *N*-acetylated residue; but for C2 the carbon chemical shift (70.21 ppm) is the one expected for a primary alcohol, while the proton chemical shift (5.92 ppm) is only consistent with an *O*-acetylation.

Table S3 – Assignment of the HSQC signals of the repeating unit of STCH_03 EPS.

| Unit/position | 1 | 2 | 3 | 4 | 5 | 6 |
| --- | --- | --- | --- | --- | --- | --- |
| A ^1^H (ppm) | 5.54 | 4.25 | 4.33 | 4.38 | 4.62 | 4.33; 4.11 |
| A ^13^C (ppm) | 97.08 | 76.93 | 68.70 | 69.66 | 69.94 | 68.70 |
| B ^1^H (ppm) | 5.43 | 4.38 | 4.18 | 3.80 | 4.02 | 1.60 |
| B ^13^C (ppm) | 99.41 | 76.79 | 69.94 | 72.27 | 70.07 | 17.16 |
| C ^1^H (ppm) | 5.33 | 5.92 | 4.18 | 3.82 | 3.77 | 1.66 |
| C ^13^C (ppm) | 99.55 | 70.21 | 76.79 | 71.31 | 72.68 | 17.16 |
| D ^1^H (ppm) | 5.27 | 4.21 | 4.01 | 4.39 | 4.21 | 4.18 |
| D ^13^C (ppm) | 95.71 | 53.90 | 79.53 | 71.17 | 69.53 | 60.34 |
| E ^1^H (ppm) | 4.84 | 3.81 | 3.94 | 4.21 | 3.96 | 4.11; 4.07 |
| E ^13^C (ppm) | 104.76 | 71.72 | 73.23 | 69.53 | 75.42 | 61.44 |
| F ^1^H (ppm) | 4.81 | 3.63 | 3.92 | 3.95 | 3.78 | 4.18; 4.04 |
| F ^13^C (ppm) | 102.84 | 73.64 | 75.83 | 72.34 | 75.01 | 61.16 |
| G ^1^H (ppm) | 4.74 | 3.82 | 4.06 | 4.29 | 4.04 | 4.09; 4.04 |
| G ^13^C (ppm) | 103.52 | 71.31 | 73.78 | 76.52 | 75.42 | 61.79 |

The acetyl group at position 2 of residue C presents a methyl group resonating at 20.86 ^13^C ppm and 2.51 ^1^H ppm with its carboxyl group generating a signal at 173.86 ^13^C ppm. The other acetyl group at position 2 of residue D generates signals at 22.37 and 174.72 ppm for ^13^C and 2.33 ppm for ^1^H.

Analysis of the coupling constants (Table S4) and chemical shift values at ring closure (position 5) allowed us to conclude that A, B and D are in the alpha pyranosil while E, F, and G are in the beta pyranosil configuration. Residue C has a carbon constant typical of the beta configuration but an anomeric chemical shift value closer to an alpha, however, in beta rhamnose residues this is not uncommon and the chemical shift value at ring closure (72.68 ppm) is a strong indicator of the beta form.

Table S4 – Coupling constants of position 1 of the repeating unit of STCH_03 EPS.

| Unit | A | B | C | D | E | F | G |
| --- | --- | --- | --- | --- | --- | --- | --- |
| ^1^J_CH1_ (Hz) | 179 | 178 | 168 | 177 | 170 | 166 | 168 |
| ^3^J_H1H2_ (Hz) | 3.3 | 2.0 | ≤ 1 | 4.0 | 7.7 | 7.8 | 7.8 |

*A value of ^1^J_CH_ above 170Hz is representative of an alpha pyranose configuration while a value between 160 and 170 is typical of the beta configuration. A value of ^3^J_H1H2_ around 3-4Hz is related to an alpha configuration, a value around 7-8 a beta configuration and a value below 2Hz is a consequence of a proton at an equatorial orientation at position 2 (the case of Rhamnose or Mannose).*

To distinguish glucose from galactose (whose sole difference is the hydroxyl orientation at position 4) we used the J-resolved proton spectrum, when possible, to measure values of ^3^J_H4_ and analysed chemical shift values at position 2 and 4 to arrive at the conclusion that A, E, and G are galactose residues, D is a glucosamine and F is glucose residue.

Recapitulating, the residues are identified as: A- alpha pyranosyl galactose; B- alpha pyranosyl rhamnose; C- beta pyranosyl 2-*O*-acetyl-rhamnose; D- alpha pyranosyl 2-*N*-acetyl-glucosamine; E- beta pyranosyl galactose; F- beta pyranosyl glucose; and G- beta pyranosyl galactose.

Figure S6 – ^13^C/^1^H HSQC spectrum of STCH_03 EPS (A) and respective expansion of the central region (B).

From the analysis of the HMBC spectrum it was possible to detect the following contacts: A1-C3, B1-G4, C1-F4, D1-B2, E1-A2, F1-D3 and G1-A6. So, the repeating unit is (Fig S7):

Figure S7 - Schematic representation of the structure of the repeating unit of STCH_03 EPS.

**STCH_06 EPS structure**

Preliminary analysis of the ^1^H spectrum revealed that the repeating unit of STCH_06 EPS was composed of 5 saccharide residues (5 signals in the anomeric region of the spectrum). The presence of a signal (a doublet at 1.62 ppm) was indicative of the presence of a rhamnose unit, while a signal at 2.35 (a singlet) was indicative of an acetylation.

A sample of STCH_06 EPS was completely hydrolyzed to its saccharide units and the monomers tentatively assigned by thin layer chromatography. ^1^H-NMR analysis of this sample revealed the presence of rhamnose, galactose, glucose glucosamine/galactosamine (not differentiated by TLC analysis), in the proportions of 1:1:2:1. The NMR identification of the monomeric units agreed with that obtained from TLC.

An array of different NMR spectra, COSY, TOCSY, ^13^C-HSQC and ^13^C-HSQC-TOCSY, integrated analysis of the data and a comparison with EPSs with similar repeating unit composition allowed the complete assignment of all the resonances (Table S5) (Fig. S8, S9, S10)

Table S5 – Assignment of the repeating unit of STCH_06 EPS.

| Unit/position | 1 | 2 | 3 | 4 | 5 | 6 |
| --- | --- | --- | --- | --- | --- | --- |
| A ^1^H (ppm) | 5.33 | 4.30 | 4.23 | 3.94 | 4.14 | 1.62 |
| A ^13^C (ppm) | 102.27 | 70.77 | 70.83 | 81.24 | 67.96 | 17.58 |
| B ^1^H (ppm) | 5.27 | 3.90 | 4.25 | 3.98 | 4.54 | 4.88; 4.10 |
| B ^13^C (ppm) | 100.34 | 72.12 | 71.59 | 78.96 | 71.18 | 60.15 |
| C ^1^H (ppm) | 5.10 | 4.24 | 4.12 | 4.35 | 4.03 | nd |
| C ^13^C (ppm) | 102.58 | 53.4 | 71.18 | 77.26 | 75.63 | nd |
| D ^1^H (ppm) | 4.85 | 3.68 | 3.97 | 3.98 | 3.92 | 4.27; 4.11 |
| D ^13^C (ppm) | 102.60 | 73.34 | 74.69 | 78.96 | 75.22 | 60.65 |
| E ^1^H (ppm) | 4.79 | 3.96 | 3.99 | 4.29 | 4.03 | nd |
| E ^13^C (ppm) | 103.23 | 70.83 | 80.60 | 68.72 | 75.63 | nd |

The acetyl group at position 2 of the glucosamine/galactosamine generates a signal at 175.39 ^13^C ppm (carboxyl group); the methyl group resonates at 22.76 ^13^C ppm and 2.35 ^1^H ppm. Due to severe overlap of positions C5 and E5 both in proton and carbon, the signals at 4.03; 4.05 ^1^H ppm/ 61.3 ^13^C ppm and 4.20; 4.16 ^1^H ppm/ 60.5 ^13^C ppm could not be unequivocally attributed to C6 and E6.

Analysis of the chemical shift values and the C-H coupling constants (Table S6) together with a comparison of NMR spectra of EPSs of strains ST144 (Vaningelgem et al 2004), NGB-22D and DS65008 (Maljaars, Van Hee 2020, EP3066223B2) possessing a similar repeating unit composition allowed to conclude that: A is a rhamnose unit in the alpha pyranosyl configuration; B is a glucose unit in the alpha pyranosyl configuration; C is a 2-N-acetyl-galactosamine in the beta pyranosyl configuration; D is a glucose unit in the beta pyranosyl configuration and E is a galactose in the alpha pyranosyl configuration.

Table S6 – ^1^J_CH_ of position 1 of the repeating unit of STCH_06 EPS.

| Unit | A | B | C | D | E |
| --- | --- | --- | --- | --- | --- |
| ^1^J_CH_ (Hz) | 172 | 170 | 160 | 160 | 159 |

*A value of ^1^J_CH_ around 170Hz is representative of an alpha configuration while a value of 160 is typical of the beta configuration.*

The acquisition and interpretation of an HMBC spectrum allowed linking the five units in the following way: B1→4C1→4A1→3E1→4D1→4. So, the repeating unit is (Fig. S11):

Figure S8 – ^13^C/^1^H HSQC spectrum of STCH_06 EPS (A) and respective expansion of the central region (B).

Figure S9 - ^13^C/^1^H HSQC spectrum of STCH_06 EPS (expansion of the CH_2_ position 6 region)

Figure S10 - ^13^C/^1^H HSQC spectra of STCH_06 (blue) and ST144 (red) EPSs.

Figure S11 - Schematic representation of the structure of the repeating unit of STCH_06 EPS. Same structure as for ST144, NGB-22D and DS65008 strains.

**STCH_07 EPS structure**

Analysis of the ^13^C HSQC spectrum (Fig. S12) revealed the presence of 7 signals in the anomeric region (corresponding to a repeating unit composed of 7 saccharide residues) and two signals in the region where methyl groups resonate. One of these methyl group signals (proton and carbon chemical shift of 1.66 and 17.3 ppm, respectively) was a doublet typical of a rhamnose residue, while the other (at 2.34 and 22.9 ppm) was indicative of an acetylation. Also, a carbon resonance at 52.7 ppm is indicative of an N substitution, leading to the hypothesis of an N-acetylated sugar residue.

Figure S12 – ^13^C HSQC spectrum of STCH_07 (multiplicity edited) at 60^o^C acquired on an AVANCE III 800 spectrometer. The signals in green are due to CH_2_ groups while the signals in blue are either CH or CH_3_ groups.

An array of different NMR spectra, COSY, TOCSY, ^13^C-HSQC and ^13^C-HSQC-TOCSY, and integrated analysis of the data did not allow the unequivocal assignment of all the resonances in the HSQC spectrum, especially due the severe signal overlap around signal D1 and the signal later identified as A5, both in carbon and in the proton frequencies. In an attempt to solve the overlap a second array of spectra was recorded at 25^o^C. The differential shifts observed in the two sets, combined with an integrated analysis of all the available data allowed the complete assignment of all the resonances (Table S7) (Fig. S12 and S13).

Table S7 – Assignment of the repeating unit of STCH_07 EPS.

| Unit/position | 1 | 2 | 3 | 4 | 5 | 6 |
| --- | --- | --- | --- | --- | --- | --- |
| A ^1^H (ppm) | 5.44 | 4.18 | 4.19 | 4.30 | 4.64 | 4.30 ;4.02 |
| A ^13^C (ppm) | 96.3 | 68.8 | 69.8 | 69.2 | 69.2 | 68.4 |
| B ^1^H (ppm) | 5.39 | 4.58 | 4.59 | 4.45 | 4.24 | 4.02 ;3.98 |
| B ^13^C (ppm) | 108.5 | 80.1 | 84.7 | 82.2 | 70.8 | 63.3 |
| C ^1^H (ppm) | 5.19 | 4.80 | 4.01 | 3.89 | 3.80 | 1.66 |
| C ^13^C (ppm) | 100.8 | 75.3 | 78.4 | 71.1 | 73.0 | 17.3 |
| D ^1^H (ppm) | 5.18 | 3.62 | 3.76 | 3.71 | 3.72 | 4.19 ;4.04 |
| D ^13^C (ppm) | 102.9 | 74.1 | 76.3 | 70.2 | 76.3 | 61.3 |
| E ^1^H (ppm) | 4.90 | 3.63 | 3.97 | 3.94 | 3.85 | 4.26 ;4.13 |
| E ^13^C (ppm) | 102.4 | 73.5 | 75.8 | 78.4 | 75.1 | 61.4 |
| F ^1^H (ppm) | 4.82 | 3.61 | 3.80 | 3.70 | 3.90 | 4.35 ;4.03 |
| F ^13^C (ppm) | 103.2 | 73.5 | 76.2 | 70.3 | 75.4 | 67.6 |
| G ^1^H (ppm) | 4.77 | 4.19 | 4.02 | 4.25 | 4.15 | 4.35 ;4.23 |
| G ^13^C (ppm) | 102.1 | 52.7 | 71.3 | 68.4 | 74.4 | 69.8 |

*The chemical shift values of the acetyl group are: Carboxilic carbon: 175 ppm, methyl group: 2.34 and 22.9 ppm in proton and carbon chemical shift, respectively.*

Chemical shift analysis shows that residue B is in the furanic form, while all the other residues are in the pyranose configuration (indeed, this residue displays chemical shift values which are typical of a galactofuramose in the beta configuration). Based on the analysis of the ^3^J_HH_ couplings at position 1 (Table S8) it can be concluded that residue A is in the alpha configuration while residues D, E, F, and G are in the beta configuration. To determine the configuration of residue C (a rhamnose) we had to measure ^1^J_CH_ at position one from the analysis of an non ^1^H decoupled ^13^C HSQC (Table S8)). A value of 164 Hz proves the beta configuration of the rhamnose residue.

Table S8 – Coupling constants at position 1 of the repeating unit of STCH_08 EPS.

| Unit | A | B | C | D | E | F | G |
| --- | --- | --- | --- | --- | --- | --- | --- |
| ^3^J_HH_ (Hz) | 3.5 | ≤2 | ≤2 | 8.0 | 7.8 | 7.9 | 8.2 |
| ^1^J_CH_ (Hz) | 176 | 184 | 164 | 169 | 168 | 169 | 166 |

*A value of ^1^J_CH_ around 170Hz is representative of an alpha configuration while a value of 160 is typical of the beta configuration.*

Figure S13 – ^13^C HSQC spectrum of STCH_07 (multiplicity edited; expansion of the central region) with the complete assignment. The signals in green are due to CH_2_ groups, while the signals in blue are either CH or CH_3_ groups.

Chemical shift analysis also indicated that positions A6, G6, and F6 and B3, C3, E4, and C2 were involved in glycosidic bonds. In fact, the acquisition and interpretation of the HMBC spectra at 60 and 25^o^C allowed linking the seven units in the following way: A1→3C1→4E1→3B1→6F1→6G1→6A, with D1 linking to position C2

At this stage a sample of STCH_07 EPS was completely hydrolyzed to its saccharide units. Based on ^1^H-NMR analysis, the hydrolysate contained glucose, galactose, rhamnose, and galactosamine (these attributions were confirmed by spiking the hydrolysate with authentic samples). At this stage we can state that: B is a beta galactofuranose, C is a beta rhamnopyranose, and G is a beta 2-*N*-acetyl-galactosamine in the pyranose form. Further analysis of the network of coupling constants and typical chemical shift values for glucose and galactose revealed that A is an alpha galactopyranose, while D, E and F are beta glucopyranoses.

Therefore, we conclude that the exopolysaccharide has the following repeating unit (Fig. S14):

Figure S14 – Schematic representation of the repeating unit of STCH_07 EPS.

**STCH_08 EPS structure**

Preliminary analysis of the ^1^H spectrum revealed that the repeating unit of STCH_08 EPS was composed of 7 saccharide residues (5 signals in the anomeric region of the spectrum with intensities 2:2:1:1:1). The presence of two doublets at 1.69 and 1.73 ppm was indicative of the presence of two rhamnose units.

A sample of STCH_08 EPS was completely hydrolyzed to its saccharide units and the monomers assigned by thin layer chromatography as rhamnose and galactose. ^1^H-NMR analysis of this sample revealed the presence of rhamnose, and galactose in the proportions of 2:5. The NMR identification of the monomeric units is in agreement with that obtained from TLC.

An array of different NMR spectra, COSY, TOCSY, ^13^C-HSQC and ^13^C-HSQC-TOCSY, and integrated analysis of the data allowed the almost complete assignment of all the resonances (Table S9) (Fig. S15)

Table S9 – Assignment of the repeating unit of STCH_08 EPS.

| Unit/position | 1 | 2 | 3 | 4 | 5 | 6 |
| --- | --- | --- | --- | --- | --- | --- |
| A ^1^H (ppm) | 5.66 | 4.41 | 4.55 | 4.57 | nd | nd |
| A ^13^C (ppm) | 93.7 | 67.3 | 75.6 | 66.9 | nd | nd |
| B ^1^H (ppm) | 5.65 | 4.45 | 4.27 | 3.88 | 4.10 | 1.69 |
| B ^13^C (ppm) | 100.6 | 79.4 | 70.5 | 72.7 | 69.9 | 17.3 |
| C ^1^H (ppm) | 5.57 | 4.36 | 4.50 | 4.44 | 4.64* | 4.15 |
| C ^13^C (ppm) | 95.8 | 74.6 | 69.9 | 70.3 | 70.9* | 61.6 |
| D ^1^H (ppm) | 5.57 | 4.44 | 4.44 | 4.61 | 4.62* | 4.15 |
| D ^13^C (ppm) | 96.3 | 67.3 | 75.6 | 66.8 | 71.5* | 61.6 |
| E ^1^H (ppm) | 5.41 | 4.75 | 4.52 | 4.40 | 4.27 | 1.73 |
| E ^13^C (ppm) | 102.4 | 65.8 | 73.6 | 76.1 | 68.7 | 17.7 |
| F ^1^H (ppm) | 5.06 | 3.91 | 4.09 | 4.36 | 4.23 | nd |
| F ^13^C (ppm) | 103.4 | 71.4 | 73.4 | 69.5 | 74.3 | nd |
| G ^1^H (ppm) | 4.86 | 3.94 | 4.05 | 4.32 | 4.07 | 4.19;4.13 |
| G ^13^C (ppm) | 103.7 | 71.4 | 73.4 | 69.3 | 75.7 | 61.7 |

Positions attributed to C5 and D5 may be exchanged. A5, A6 and F6 could not be assigned.

Analysis of the chemical shift values and the C-H coupling constants derived from the analysis of a non ^1^H decoupled ^13^C HSQC (Table S9) allowed us to conclude that: A, C and D are galactose units in the alpha pyranosyl configuration; B and E are rhamnose units in the alpha pyranosyl configuration; and F and G are galactose units in the beta pyranosyl configuration.

Table S9 – ^1^J_CH_ of position 1 of the repeating unit of STCH_08 EPS.

| Unit | A | B | C | D | E | F | G |
| --- | --- | --- | --- | --- | --- | --- | --- |
| ^1^J_CH_ (Hz) | 175 | 175 | 170 | 171 | 170 | 166 | 163 |

*A value of ^1^J_CH_ around 170Hz is representative of an alpha configuration while a value of 160 is typical of the beta configuration.*

The acquisition and interpretation of an HMBC spectrum allowed linking four of the seven units in the following way: F1→4E1→2B1→2C1, but due to severe overlap in both proton and carbon frequencies and to low intensity of the HMBC signals, no other glycosidic linkages could be established.

Since our data aligned with the findings of Bubb et al. (1997) (Fig. S16), we concluded that STCH_08 produces an exopolysaccharide with the same repeating unit as described by Bubb et al. (Fig. S17)

Figure S15 –^13^C/^1^H HSQC spectrum of STCH_08 EPS (A) and respective expansion of the central region (B).

Figure S16 – Comparison between the HSQC spectra of STCH_08 EPS (left panels) and the EPS from Bubb et al. 1997 (right panels).

Figure S17 – Schematic representation of the repeating unit of STCH_08 EPS.

**STCH_09 EPS structure**

Preliminary analysis of the ^1^H spectrum revealed that the repeating unit STCH_09 EPS was composed of 4 saccharide residues (4 signals in the anomeric region of the spectrum). No methyl signals were present, ruling out the possibility of rhamnose or acetylated carbohydrate units.

A sample of STCH_09 EPS was completely hydrolyzed to its saccharide units and the monomers tentatively assigned by thin layer chromatography. ^1^H-NMR analysis of this sample revealed the presence of galactose and glucose in the proportions of 1:1. The NMR identification of the monomeric units is consistent with the results obtained from TLC.

An array of different NMR spectra, COSY, ^13^C-HSQC and ^13^C-HSQC-TOCSY, and integrated analysis of the data allowed the complete assignment of all the resonances (Table S10) (Fig. S18)

Table S10 – Assignment of the repeating unit of STCH_09 EPS.

| Unit/position | 1 | 2 | 3 | 4 | 5 | 6 |
| --- | --- | --- | --- | --- | --- | --- |
| A ^1^H (ppm) | 5.64 | 4.00 | 4.18 | 3.81 | 4.34 | 4.12; 4.10 |
| A ^13^C (ppm) | 99.49 | 72.01 | 80.35 | 68.33 | 72.22 | 60.94 |
| B ^1^H (ppm) | 5.61 | 4.67 | 4.61 | 4.56 | 4.28 | 4.02; 3.98 |
| B ^13^C (ppm) | 108.92 | 80.22 | 85.00 | 82.68 | 70.85 | 63.47 |
| C ^1^H (ppm) | 4.96 | 3.74 | 3.98 | 4.04 | 3.97 | 4.51; 4.20 |
| C ^13^C (ppm) | 102.56 | 72.15 | 82.68 | 70.24 | 75.16 | 68.94 |
| D ^1^H (ppm) | 4.74 | 3.88 | 3.95 | 4.24 | 3.99 | 4.10; 4.08 |
| D ^13^C (ppm) | 103.93 | 71.33 | 73.25 | 69.15 | 75.57 | 61.42 |

STCH_09 EPS is composed solely of glucose and galactose residues. The only difference between these two carbohydrates is at position 4. While glucose has all its CH protons in the axial orientation in respect to the plane of the ring, galactose has its proton 4 in an equatorial orientation. The most obvious spectroscopical consequence of this difference is the values of ^3^J_HH_ constants. Axial-axial proton pairs give rise to 9-12Hz constants while axial-equatorial produces 2-6Hz and equatorial-equatorial 2-3Hz.

Analysis of the chemical shift values and the available coupling constants allowed us to conclude that: B and D are galactose residues because the proton coupling constants on position 4 are between 4 and 5 Hz; position 4 of residue A displays a triplet with a 9.8Hz constant which allows us to conclude that it is a glucose; position 4 of residue C is overlapping other ressonances and its proton coupling constants could not be determined, but since we have only glucose and galactose in a 1:1 proportion, C has to be a glucose residue

The alpha or beta configuration of the carbohydrate ring determines the orientation of proton 1, which in turn, has consequences both in the chemical shifts and the coupling constants. 1) ^1^J_CH_ – in pyranoses alpha configurations give rise to values around or above 170Hz, while beta configuration results in values around 160Hz; 2) chemical shifts – an alpha configuration results in higher chemical shift values for proton and lower for carbon; 3) ^3^J_HH_ – the coupling constant between proton 1 and proton 2 in pyranoses is around 3Hz or 7Hz for alpha or beta respectively.

A review of the measured coupling constants (Table S11) clearly indicates that glucose A is in the alpha configuration, while glucose C and galactose D are in the beta. Galactose B however has a ^3^J_HH_ coupling constant, which is impossible for galactopyranose, but could be consistent with galactofuranose. In fact, the chemical shift values are consistent with a beta galactofuranose configuration as reported by Lemoine et al (1997).

Table S11 – Coupling constants of position 1 of the repeating unit of STCH_09 EPS*

| Unit | A | B | C | D |
| --- | --- | --- | --- | --- |
| ^1^J_CH_ (Hz) | 176 | 178 | 164 | 165 |
| ^3^J_HH_ (Hz) | 4.4 | ≤2 | 7.9 | 7.7 |

*^1^J_CH_ constants were obtained from a non-decoupled 13C HSQC while ^3^J_HH_ were measured from a H-H J-resolved 2D spectrum.

Figure S18 –^13^C/^1^H HSQC spectrum of STCH_09 EPS (A) and respective expansion of the central region (B).

The acquisition and interpretation of an HMBC spectrum allowed linking the four units in the following way: A1→3C1→3B1→3A and a ramification at position 6 of the C unit D1→6C. So, the repeating unit is (Fig. S19):

Figure S19 – Structural representation of the repeating unit of STCH_09 EPS.

**STCH_10 EPS structure**

Preliminary analysis of the ^1^H spectrum revealed that the repeating unit of STCH_10 EPS was composed of 6 saccharide residues (6 signals in the anomeric region of the spectrum). The presence of two doublets at 1.46 and 1.48 ppm was indicative of the presence of two rhamnose units, while a signal at 2.19 (a singlet) was indicative of an acetylation.

A sample of STCH_10 EPS was completely hydrolyzed to its saccharide units and the monomers assigned by ^1^H-NMR with spiking of authentic samples. The analysis revealed the presence of rhamnose, glucosamine, galactose and glucose.

An array of different NMR spectra, COSY, ^13^C-HSQC and ^13^C-HSQC-TOCSY, and integrated analysis of the data allowed the complete assignment of all the resonances (Table S12) (Fig S20)

Table S12 – assignment of the repeating unit of STCH_10 EPS.

| Unit/position | 1 | 2 | 3 | 4 | 5 | 6 |
| --- | --- | --- | --- | --- | --- | --- |
| A ^1^H (ppm) | 5.28 | 4.23; | 4.03; | 3.66; | 3.88; | 1.46; |
| A ^13^C (ppm) | 99.15 | 76.41 | 69.61 | 73.07 | 69.85 | 16.98 |
| B ^1^H (ppm) | 5.27 | 4.00 | 4.09 | 4.21 | 4.51 | 4.19;3.95 |
| B ^13^C (ppm) | 96.34 | 68.44 | 69.38 | 69.38 | 69.73 | 68.68 |
| C ^1^H (ppm) | 5.12 | 4.07 | 4.07 | 3.88 | 4.26 | 4.03 |
| C ^13^C (ppm) | 95.52 | 53.67 | 69.26 | 79.34 | 71.02 | 60.12 |
| D ^1^H (ppm) | 5.01 | 4.42 | 3.83 | 3.63 | 3.57 | 1.48 |
| D ^13^C (ppm) | 100.68 | 67.74 | 78.40 | 70.67 | 72.31 | 17.10 |
| E ^1^H (ppm) | 4.68 | 3.49 | 3.81 | 3.81 | 3.70 | 4.07;3.97 |
| E ^13^C (ppm) | 102.78 | 73.48 | 75.59 | 76.88 | 74.89 | 60.94 |
| F ^1^H (ppm) | 4.59 | 3.68 | 3.91 | 4.13 | 3.90 | 3.96; 3.88 |
| F ^13^C (ppm) | 103.37 | 71.02 | 73.60 | 76.29 | 75.12 | 61.64 |

The acetyl group at position 2 of the glucosamine generates a signal at 174.24 ^13^C ppm (carboxyl group); the methyl group resonates at 22.14 ^13^C ppm and 2.19 ^1^H ppm.

Analysis of the chemical shift values allowed us to conclude that we are in the presence of pyranose rings (furanoses display ^13^C chemical shifts at ring closure between 80 and 86 ppm). The ^13^C chemical shift at ring closure (position 5 in the case of aldo-pyranoses) is also indicative of beta or alpha configuration since beta anomers resonate at higher ppm values (around 75 ppm) than alpha anomers (around 71 ppm). In addition, the proton chemical shift value at position 1 is higher for the alpha than for the beta pyranoses. That information, combined with the C-H, and H-H coupling constants at position 1 (Table S13) allowed us to conclude that: A is a rhamnose unit in the alpha configuration; B is a galactose or glucose unit in the alpha configuration; C is a 2-N-acetyl-glucoseamine in the alpha configuration; D is a rhamnose unit in the beta configuration and E and F are glucose or galactose units in the beta configuration.

At this point, the only ambiguity lies in the identity or residues B, E and F, which can be either glucose or galactose residues. The only structural difference between these two carbohydrates lies in position 4. While glucose has all its CH protons in the axial orientation in respect to the plane of the ring, galactose has its proton 4 in an equatorial orientation. The most obvious spectroscopical consequence of this difference is the values of ^3^J_HH_ constants. Axial-axial proton pairs give rise to 9-12Hz constants while axial-equatorial produces 2-6Hz and equatorial-equatorial 2-3Hz.

Even with the acquisition of a J-resolved spectrum, not all the constants on position 4 could be measured, however, in residues B and F, constants in the order of 4-5 Hz could be measured at position 4, meaning that these residues are galactoses. Regarding residue E, a clear measurement was not possible, however, its chemical shift patterns are consistent with a beta glucose, and this carbohydrate was present in the hydrolysate, therefore residue E must be a glucose residue.

Table S13 – Coupling constants of position 1 of the repeating unit of STCH_10 EPS.

| Unit | A | B | C | D | E | F |
| --- | --- | --- | --- | --- | --- | --- |
| ^1^J_CH_ (Hz) | 173 | 174 | 175 | 165 | 168 | 169 |
| ^3^J_H1H2_ (Hz) | ~1.5 | 3.8 | 3.6 | ~1.5 | 7.9 | 7.8 |

*A value of ^1^J_CH_ around or above 170Hz is representative of an alpha pyranose configuration while a value between 160 and 170 is typical of the beta configuration. A value of 3JH1H2 around 4Hz is raelated to an alpha configuration, a value around 7.5 a beta configuration and a value below 2Hz is a consequence of a proton at an equatorial orientation at position 2 (the case of rhamnose or mannose)*

Figure S20 –^13^C/^1^H HSQC spectrum of STCH_10 EPS (A) and respective expansion of the central region (B).

The acquisition and interpretation of an HMBC spectrum allowed linking the six units in the following way: A1→4F1→6B1→3D1→4E1→4C1→2A. Therefore, the repeating unit is (Fig. S21):

Figure S21 – Structural representation of the repeating unit of STCH_10 EPS.

**STCH_01 EPS and STCH_05 EPS structures**

Initial analysis of the NMR spectra from STCH_01 EPS and STCH_05 EPSs revealed striking similarities. In fact, the repeating unit of STCH_05 EPS differs from STCH_01 EPS only by the addition of a single carbohydrate residue (see below). Therefore, the conclusions from one set of spectra could be used to interpret the other set and vice-versa, thus finding an additional way of trying to escape the very severe overlap of the measured proton and carbon frequencies.

Preliminary analysis of the ^1^H spectrum of STCH_01 EPS revealed a repeating unit composed of 5 saccharide residues (5 signals in the anomeric region of the spectrum). One of these residues presents a H-H coupling constant that is not measurable in the 1D spectrum, which suggested the presence of a mannose residue. However, a 2D J-resolved spectrum of this sample allowed measuring a constant of 3.6 Hz – indicative of an alpha configuration of the sugar residue. The other four signals presented constants around 7 Hz indicative of residues in the beta configuration (probably glucose or galactose). No methyl signals were present, ruling out the possibility of rhamnose or acetylated carbohydrate units.

The ^1^H spectrum of STCH_05 EPS revealed a repeating unit composed of 6 saccharide residues, 5 of which were strikingly similar to the ones found in STCH_01 EPS. In fact, a superimposition of ^13^C-HSQC spectra from both EPS’s showed an almost complete identity with additional signals from the extra carbohydrate residue. The additional anomeric signal is a doublet with a H-H coupling constant of 7.8 Hz (probably glucose or galactose in the beta configuration).

A sample of STCH_01 EPS was hydrolyzed in order to identify its saccharide units. Using thin layer chromatography only one spot was identified (the other constituents were obscured in a smear) that migrated with mannose, however, the solvent system used made it difficult to distinguish mannose from glucose. Through g ^1^H-NMR analysis the monosaccharides glucose (glc) and galactose (gal) were identified by spiking this sample with the pure compounds and mannose could not be detected. The relative intensities of the glc and gal residues was 3:1.88, indicating a repeating unit composed of three glucose and two galactose residues. The analysis of the spectra of STCH_01 EPS (see below) showed that four carbohydrate residues in the beta configuration could be divided into two patterns of ^13^C and ^1^H chemical shifts, consistent with beta glucose and beta galactose. So, the remaining residue must be a glucose in the alpha configuration.

Since five of units STCH_01 EPS were identical in STCH_05 EPS, only the extra residue required identification. So, a sample of STCH_05 EPS was hydrolysed and analysed by TLC and NMR. The NMR revealed the presence of glucose and galactose in the relative proportions of 4:2.17, indicationg an extra glucose in the beta configuration.

An array of different NMR spectra, COSY, TOCSY, ^13^C-HSQC and ^13^C-HSQC-TOCSY, were acquired for both STCH_01 and STCH_05 EPS’s. An integrated analysis of the two data sets allowed the complete assignment of almost all the resonances (Tables S14 and S15) (Figs. S22 and S23) ). Due to severe overlap, positions E5, D5 and most of the signals belonging to positions 6 cannot be unequivocally attributed.

Residue A was identified as a glucose in the alpha configuration. The other four residues were glucose and galactose in the beta configuration. The chemical shift patterns allowed us to conclude that residues B and C were glucose, while D and E were galactose residues.

Table S14 – Assignment of the repeating unit of STCH_01 EPS.

| Unit/position | 1 | 2 | 3 | 4 | 5 | 6 |
| --- | --- | --- | --- | --- | --- | --- |
| A ^1^H (ppm) | 5.22 | 3.90 | 4.15 | 4.06 | 4.66 | 4.46; 4.27 |
| A ^13^C (ppm) | 100.21 | 72.02 | 71.67 | 78.71 | 70.10 | 67.77 |
| B ^1^H (ppm) | 4.90 | 3.67 | 3.96 | 3.94 | 3.91 | nd |
| B ^13^C (ppm) | 102.61 | 73.36 | 74.79 | 79.19 | 75.23 | nd |
| C ^1^H (ppm) | 4.82 | 3.68 | 3.96 | 3.94 | 3.89 | 4.28; 4.12 |
| C ^13^C (ppm) | 102.75 | 73.25 | 74.79 | 79.16 | 75.16 | 60.73 |
| D ^1^H (ppm) | 4.80 | 3.89 | 4.04 | 4.33 | nd | nd |
| D ^13^C (ppm) | 103.68 | 71.33 | 72.60 | 77.82 | nd | nd |
| E ^1^H (ppm) | 4.75 | 3.85 | 3.95 | 4.23 | nd | nd |
| E ^13^C (ppm) | 103.41 | 71.47 | 73.12 | 69.08 | nd | nd |

nd-not determined

Figure S22 –^13^C/^1^H HSQC spectrum of STCH_01 EPS (A) and respective expansion of the central region (B). 5* and 5’ correspond to position 5 of either residue D or E; 6* correspond to position 6 of the same residue as 5*.

Table S15 – assignment of the repeating unit of STCH_05 EPS.

| Unit/position | 1 | 2 | 3 | 4 | 5 | 6 |
| --- | --- | --- | --- | --- | --- | --- |
| A ^1^H (ppm) | 5.21 | 3.90 | 4.16 | 4.13 | 4.65 | 4.44; 4.32 |
| A ^13^C (ppm) | 100.2 | 71.97 | 71.73 | 79.79 | 69.95 | 67.76 |
| B ^1^H (ppm) | 4.89 | 3.68 | 3.96 | 4.01 | 3.93 | nd |
| B ^13^C (ppm) | 103.06 | 73.26 | 74.74 | 79.09 | 75.24 | nd |
| C ^1^H (ppm) | 4.95 | 3.97 | 4.19 | 3.99 | 3.90 | nd |
| C ^13^C (ppm) | 101.43 | 80.28 | 75.53 | 78.71 | 75.07 | nd |
| D ^1^H (ppm) | 4.80 | 3.90 | 4.04 | 4.33 | nd | nd |
| D ^13^C (ppm) | 103.77 | 71.35 | 72.59 | 77.76 | nd | nd |
| E ^1^H (ppm) | 4.75 | 3.86 | 3.96 | 4.25 | nd | nd |
| E ^13^C (ppm) | 103.32 | 71.48 | 73.09 | 69.12 | nd | nd |
| F ^1^H (ppm) | 5.12 | 3.63 | 3.82 | 3.64 | 3.74 | 4.22; 4.02 |
| F ^13^C (ppm) | 103.31 | 74.50 | 76.44 | 70.49 | 76.73 | 61.72 |

nd-not determined

Figure S23 –^13^C/^1^H HSQC spectrum of STCH_05 EPS (A) and respective expansion of the central region (B). 5* and 5’ correspond to position 5 of either residue D or E; 6* correspond to position 6 of the same residue as 5*.

The acquisition and interpretation of HMBC spectra allowed linking the units. In EPS2389 the following correlations could be found: A1→D4; B1→A4; C1→A6 and E1,D1→C4,B4. The overlapping signals led to the ambiguity regarding which residue E or D was linking to the position 4 of B or C. In EPS5389 only the A1→D4, E1→B4 and F1→B2 correlations were detected, however since both EPS’s share the same 5 residue core structure, the following was concluded (Fig. S25, Fig. S26):

Figure S25 – Schematic representation of the structure of the repeating unit of STCH_01 EPS.

Figure S26 – Schematic representation of the structure of the repeating unit of STCH_05 EPS.

# **Accession numbers for *eps* clusters**

| **Strain** | **NCBI accession number** |
| --- | --- |
| *Lactobacillus delbueckii* *subsp. bulgaricus* Lfi5 | AF267127 |
| *Lactobacillus fermentum* TDS030603 | AB519644 |
| *Lactobacillus helveticus* NCC2745 | AX009415 |
| *Lactococcus lactis* B40 | AF036485 |
| *Lactococcus lactis* B891 | AF100298 |
| *Lb. helveticus* MB2-1 | CP011386 |
| *Lb. paracasei* ZY-1 | CP065154 |
| *Lb. plantarum* TMW 1.1478 | CP021932 |
| *Lb. rhamnosus* GG | NC_013198 |
| *S. thermophilus* ASCC 1275 | CP006819 |
| *S. thermophilus* EU20 | DQ393660 |
| *S. thermophilus* LY03 | DQ393658 |
| *S. thermophilus* MR-1C | AF448249 |
| *S. thermophilus* Sfi39 | AF373595 |
| *S. thermophilus* Sfi6 | U40830 |
| *S. thermophilus* STCH_01 | PV446625 |
| *S. thermophilus* STCH_02 | MK483553 |
| *S. thermophilus* STCH_03 | PV446626 |
| *S. thermophilus* STCH_04 | PV446624 |
| *S. thermophilus* STCH_05 | PV446623 |
| *S. thermophilus* STCH_06 | MK483540 |
| *S. thermophilus* STCH_07 | MK483563 |
| *S. thermophilus* STCH_08 | PV446627 |
| *S. thermophilus* STCH_09 | MK483530 |
| *S. thermophilus* STCH_10 | PV446628 |

# **References**

Doco T, Wieruszeski JM, Fournet B, Carcano D, Ramos P, Loones A. (1990) Structure of an exocellular polysaccharide produced by *Streptococcus thermophilus*. Carbohydr Res. 198:313–321.

Stingele F, Neeser JR, Mollet B. (1996) Identification and characterization of the eps (Exopolysaccharide) gene cluster from *Streptococcus thermophilus* Sfi6. J Bacteriol. 178:1680-90.

Vaningelgem F, Zamfir M, Mozzi F, Adriany T, Vancanneyt M, Swings J, De Vuyst L. (2004) Biodiversity of exopolysaccharides produced by *Streptococcus thermophilus* strains is reflected in their production and their molecular and functional characteristics. Appl Environ Microbiology. 40: 900–912

Maljaars C, Van Hee P. (2020) HETEROPOLYSACCHARIDES. Patent EP3066223B2

Bubb WA, Urashima T, Fujiwara R, Shinnai T, Ariga H. (1997) Structural characterisation of the exocellular polysaccharide produced by *Streptococcus thermophilus* OR 901. Carbohydr Res. 301:41-50.

Lemoine J, Chirat F, Wieruszeski J M , Strecker G, Favre N, Neeser J R (1997) Structural characterization of the exocellular polysaccharides produced by *Streptococcus thermophilus* SFi39 and SFi12. Appl Environ Microbiol. 63:3512-3518.

# **Code and data**

Code and data for performing the analyses can be accessed on Mendeley Data under the DOI 10.17632/6w3p65cjkk.1

<https://data.mendeley.com/datasets/6w3p65cjkk/1>
